# Supplementary material for: Genome-wide association analysis of anthracnose resistance in the Yellow Bean Collection of Common Bean
Source: PLoS One. 2023 Nov 10;18(11):e0293291. doi: 10.1371/journal.pone.0293291 (PMC10637669; doi:10.1371/journal.pone.0293291)
Supplement: Attachment — Submitted filename: PONE-D-23-01331_R1-Reviews copy.pdf [file pone.0293291.s004.pdf]

PLOS ONE

Genome-wide association analysis of anthracnose resistance in the Yellow Bean Collection of Common Bean

--Manuscript Draft--

**Manuscript Number:**	PONE-D-23-01331R1
**Article Type:**	Research Article
**Full Title:**	Genome-wide association analysis of anthracnose resistance in the Yellow Bean Collection of Common Bean
**Short Title:**	Genome-wide association analysis of anthracnose resistance in the Yellow Bean Collection of Common Bean
**Corresponding Author:**	kelvin kamfwa<br>University of Zambia School of Agricultural Sciences<br>Lusaka, ZAMBIA
**Keywords:**	anthracnose; Race; genome-wide association analysis; candidate gene
**Abstract:**	Anthracnose caused by Colletotrichum lindemuthianum is a major disease of common bean (Phaseolus vulgaris) worldwide. Yellow beans are a major market class of common bean especially in eastern and southern Africa. The objectives of this study were i) evaluate the yellow bean collection of 255 genotypes for resistance to races 5, 19, 39, 51, 81, 183, 1050 and 1105 of C. lindemuthianum., and ii) conduct genome-wide association analysis to identify genomic regions and candidate genes associated with resistance to C. lindemuthianum. The Yellow Bean Collection was genotyped with 72,866 SNPs using Genotyping by Sequencing, and genome-wide association analysis was conducted using Mixed Linear Model in TASSEL. Andean and Middle American genotypes with superior levels of resistance to the eight races used in the current study were identified. The genotype YBC278 was the only one among 255 genotypes that was highly resistant to all eight races. Resistance in the Yellow Bean Collection to the eight races used in the current study was controlled by major-effect loci on chromosomes Pv01, Pv03, Pv04, Pv05 and Pv07. The genomic region on Pv01, which overlapped with the Andean locus Co-1 provided resistance to races 81, 1050 and 1105. Significant SNPs for resistance to race 39 were identified on Pv02. The genomic region on Pv04, which overlapped with known major-effect loci Co-3, Co-15, Co-16, Co-y and Co-z, provided resistance to races 5, 19, 51 and 183. Novel genomic regions for resistance to race 39 were identified on Pv05 and Pv07. Plant resistance genes (R genes) with NB-ARC and LRR domains, which occurred in clusters, were identified as positional candidate genes for genomic regions on Pv02 and Pv04.
**Order of Authors:**	kelvin kamfwa
	Kuwabo Kuwabo
	Swivia Hamabwe
	Paul Kachapulula
	Chikoti Mukuma
	Travis Parker
	Karen Cichy
**Response to Reviewers:**	Responses to Reviewer Comments<br><br>Reviewer # 1<br>Comment: The work done in this study refers to a very important biotic constraint in common bean production and some useful information has been generated in this study but authors have failed to signify the importance and novelty of this work as the MS resistance has been written as routine presentation of the data generated in the study.<br>Response: This is the first Genome-wide Association Study (GWAS) on common bean anthracnose conducted using the yellow bean diversity panel. The study has provided important and novel information on the genetic architecture of anthracnose resistance

in yellow beans, which is a major market class of common bean. In addition to confirming previously identified genomic regions for anthracnose (ANTH) resistance, the study has reported novel genomic regions for ANTH resistance. The genomic regions on chromosomes Pv05 and Pv07 that provided resistance to race 39 are novel and reported for the first time in this manuscript. The other novelty is in the races used in the current study. For the majority of these races, it is the first time the genomic regions providing resistance to them has been established, and clusters of resistance genes associated with these specific races identified. Furthermore, this study has identified a yellow bean genotype YBC278 that is highly resistant to ANTH. This is a key finding with potentially major impact on the bean breeding community working on developing yellow bean varieties resistant to ANTH. There is no previous report of a yellow bean genotype that has higher ANTH resistance than YBC278 identified in the current study. In addition to the “standard” presentation of GWAS results in the form of Manhattan plots and tables showing genomic regions associated with resistance used in the current, the manuscript has gone further to better understand the positional candidate genes underlying the significant genomic regions. The study has determined that some of the positional candidate genes underlying the identified resistance loci occur in clusters, which could possibly explain why some of these resistance loci provide resistance to multiple races of C. lindemuthianum.

Comment: The methodology details of this work are detailed in reference 4 (Genet Resour Crop Evol. 2022; 69(4): 1627-1648) cited in this study. Only citation of reference to qualify the methodology used is not sufficient to help the reader and signify the work done, hence a gist of the protocol or procedures used need mention in the material and method section.
Response: We have revised the sections, Plant materials, Genotypic data analyses and Population structure under Materials and Methods that hopefully addresses this comment.

Comment: The presentation of the data in figures is very complex and is not able to access the descriptions of the results, hence need proper attention to depict the results in figures.
Response: Results for this GWAS have been presented in a “standard” way, which involves use of histograms and tables to summarize the phenotypic data, and identify patterns in the data. Further, GWAS results have been presented using Manhattan plots and tables to show genomic regions associated with resistance to the eight races used in the current study. These tables, histograms and Manhattan plots were used to ensure that presentations of results are not complicated, and followed a “standard” way of presenting GWAS results.

Comment: Almost more than 95% of the genotypes were susceptible to most of the races. it's not clear that what were the basis of selecting the panel of genotype in this study need clarity.
Response: A number of considerations went into assembling the panel and its choice for use in the current study. First, the panel was assembled to specifically focus on yellow beans. The second consideration was that the panel had to have rich diversity of yellow bean genotypes and this was actualized by ensuring that the panel was comprised of yellow bean genotypes from breeding programs from different parts of the world. Yes, 95% of materials might have been susceptible to most races, but genotypes (few) with superior resistance (Table 2) comparable to that of even the resistant check for some races were identified. This demonstrated the importance and novelty of the study, because the identified resistant genotypes, few as they may be, are crucially important as novel sources of resistance to breed for improved ANTH resistance in yellow bean.

Comment: There are few more observations in the reviewed copy of the MS, which can improve the quality of the work done for better resolution of the results and facilitate the reader to come to conclusion of the study.
Response: Thank you for the observations. The manuscript has been revised to include all the observations that were in the reviewed copy of the manuscript.

Comment: The results are not properly described. There is no mention of the SNPs that can help in future study and likewise other output issues.
Response: We have revised the Results section to include the name of the SNPs

Powered by Editorial Manager® and ProduXion Manager® from Aries Systems Corporation

associated with resistance to races used in the current study. The SNPs are mentioned on pages 17, 18 and 19 under Results. Furthermore, the SNP names and positions have been provided in Table 3. The reason we did not provide the SNP names initially was because we thought presenting positions or genomic regions significantly associated with resistance was more useful and could facilitate future comparisons of genomic regions identified in the current study with future studies. Names of SNPs do change depending on the genotyping platform used, but the positions do not change or change very slightly when there is a new version of the genome.

Reviewer # 2
Comment: The manuscript is well written and finding a few novel anthracnose associated SNPs on PV 05 and Pv07 are of interest.
Response: Thank you.

Comment: There are a few typos that need due attention and are listed below
Response: The typo's have been corrected.

Comment: A few important references are missing from the manuscript.
Response: Several missing key references have been added to the revised manuscript.

Comment: L68-69. There is typo. Delete of and insert, hey?
Response: It was a typo and it has been corrected.

Comment: L86. ANTH may be replaced with anthracnose
Response: Replaced.

Comment: L89-91. Recently Nabi et al., 2022 have compiled this information. Not few QRLs now many have been identified do check (Simson et al., 2022; Banoo et al., 2020).
Response: Corrected and the three references have been added.

Comment: L111-112. Please do rewrite it.
Response: The sentence has been rewritten.

Comment: L117. Yellow bean Collection (YBC). Do write this acronym before also and define it in the first paragraph.
Response: Corrected

Comment: L132-134. Do rewrite it
Response: The sentence has been rewritten.

Comment: L135. Delete effect.
Response: Corrected.

Comment: L155 and L166 [28] may be replaced with Mungalu et al., (2020) as author year style.
Response: Corrected.

Comment: L168-169. Sub-script the magnesium sulphate and potassium dihydrogen othrophosphate
Response: Corrected.

Comment: L176 do provide reference at the end
Response: The reference has been added.

Comment: L206 and 212 [4] write the reference in author (year) style
Response: The author style has been changed.

Comment: L435. Do add a recent reference (Lima et al., 2023) that validated this gene model for anthracnose resistance. There are numerous GWAS studies that indicate association of this region with multiple anthracnose resistance. Do add write of such reference to support your conclusion

Powered by Editorial Manager® and ProduXion Manager® from Aries Systems Corporation

	Response: The suggested reference has been added.<br><br>Comment: L467. Add a few more references<br>Response: References have been added.<br><br>Comment: L492. Banoo et al also found SNP on PV05 and 07 against race 87 and 73. Do add that also.<br>Response: The suggested reference has been added.
**Additional Information:**	
**Question**	**Response**
**Financial Disclosure**<br><br>Enter a financial disclosure statement that describes the sources of funding for the work included in this submission. Review the submission guidelines for detailed requirements. View published research articles from *PLOS ONE* for specific examples.<br><br>This statement is required for submission and **will appear in the published article** if the submission is accepted. Please make sure it is accurate.<br><br>**Unfunded studies**<br>Enter: *The author(s) received no specific funding for this work.*<br><br>**Funded studies**<br>Enter a statement with the following details:<br>• Initials of the authors who received each award<br>• Grant numbers awarded to each author<br>• The full name of each funder<br>• URL of each funder website<br>• Did the sponsors or funders play any role in the study design, data collection and analysis, decision to publish, or preparation of the manuscript?<br>• **NO** - Include this sentence at the end of your statement: *The funders had no role in study design, data collection and analysis, decision to publish, or preparation of the manuscript.*<br>• **YES** - Specify the role(s) played.<br><br>* typeset	KK received scholarship from the Kirkhouse Trust of the United Kingdom.<br>URL:https://www.kirkhousetrust.org<br><br>The funders had no role in study design, data collection and analysis, decision to publish, or preparation of the manuscript
**Competing Interests**<br><br>Use the instructions below to enter a competing interest statement for this	The authors have declared that no competing interests exist.

Powered by Editorial Manager® and ProduXion Manager® from Aries Systems Corporation

submission. On behalf of all authors, disclose any competing interests that could be perceived to bias this work—acknowledging all financial support and any other relevant financial or non-financial competing interests.<br><br>This statement is required for submission and **will appear in the published article** if the submission is accepted. Please make sure it is accurate and that any funding sources listed in your Funding Information later in the submission form are also declared in your Financial Disclosure statement.<br><br>View published research articles from *PLOS ONE* for specific examples.<br><br>**NO authors have competing interests**<br><br>Enter: *The authors have declared that no competing interests exist.*<br><br>**Authors with competing interests**<br><br>Enter competing interest details beginning with this statement:<br><br>*I have read the journal's policy and the authors of this manuscript have the following competing interests: [insert competing interests here]*<br><br>* typeset	
**Ethics Statement**<br><br>Enter an ethics statement for this submission. This statement is required if the study involved:<br><br>• Human participants<br>• Human specimens or tissue<br>• Vertebrate animals or cephalopods<br>• Vertebrate embryos or tissues<br>• Field research<br><br>Write "N/A" if the submission does not	N/A

require an ethics statement.<br><br>General guidance is provided below. Consult the submission guidelines for detailed instructions. **Make sure that all information entered here is included in the Methods section of the manuscript.**<br><br>**Format for specific study types**<br><br>**Human Subject Research (involving human participants and/or tissue)**<br>• Give the name of the institutional review board or ethics committee that approved the study<br>• Include the approval number and/or a statement indicating approval of this research<br>• Indicate the form of consent obtained (written/oral) or the reason that consent was not obtained (e.g. the data were analyzed anonymously)<br><br>**Animal Research (involving vertebrate animals, embryos or tissues)**<br>• Provide the name of the Institutional Animal Care and Use Committee (IACUC) or other relevant ethics board that reviewed the study protocol, and indicate whether they approved this research or granted a formal waiver of ethical approval<br>• Include an approval number if one was obtained<br>• If the study involved *non-human primates*, add *additional details* about animal welfare and steps taken to ameliorate suffering<br>• If anesthesia, euthanasia, or any kind of animal sacrifice is part of the study, include briefly which substances and/or methods were applied<br><br>**Field Research**<br><br>Include the following details if this study involves the collection of plant, animal, or other materials from a natural setting:<br>• Field permit number<br>• Name of the institution or relevant body that granted permission	
**Data Availability**	Yes - all data are fully available without restriction

Authors are required to make all data underlying the findings described fully available, without restriction, and from the time of publication. PLOS allows rare exceptions to address legal and ethical concerns. See the PLOS Data Policy and FAQ for detailed information.<br><br>A Data Availability Statement describing where the data can be found is required at submission. Your answers to this question constitute the Data Availability Statement and **will be published in the article**, if accepted.<br><br>**Important:** Stating 'data available on request from the author' is not sufficient. If your data are only available upon request, select 'No' for the first question and explain your exceptional situation in the text box.<br><br>Do the authors confirm that all data underlying the findings described in their manuscript are fully available without restriction?	
**Describe where the data may be found in full sentences. If you are copying our sample text, replace any instances of XXX with the appropriate details.**<br><br>• If the data are **held or will be held in a public repository**, include URLs, accession numbers or DOIs. If this information will only be available after acceptance, indicate this by ticking the box below. For example: *All XXX files are available from the XXX database (accession number(s) XXX, XXX.*).<br>• If the data are all contained **within the manuscript and/or Supporting Information files**, enter the following: *All relevant data are within the manuscript and its Supporting Information files.*<br>• If neither of these applies but you are able to provide **details of access elsewhere**, with or without limitations, please do so. For example:	All relevant data are within the manuscript and its Supporting Information files

Powered by Editorial Manager® and ProduXion Manager® from Aries Systems Corporation

Data cannot be shared publicly because of [XXX]. Data are available from the XXX Institutional Data Access / Ethics Committee (contact via XXX) for researchers who meet the criteria for access to confidential data.<br><br>*The data underlying the results presented in the study are available from (include the name of the third party and contact information or URL).*<br>• This text is appropriate if the data are owned by a third party and authors do not have permission to share the data.<br><br>* typeset	
Additional data availability information:	

Powered by Editorial Manager® and ProduXion Manager® from Aries Systems Corporation

Manuscript Click here to access/download;Manuscript;Manuscript.docx

Genome-wide association analysis of anthracnose resistance in the Yellow Bean Collection of Common Bean

Kuwabo Kuwabo[1], Swivia M. Hamabwe[1], Paul Kachapulula[1], Karen Cichy[2], Travis Parker[3], Chikoti Mukuma[4] and Kelvin Kamfwa[1*]

[1]Dept. of Plant Sciences, University of Zambia, Great East Road, Lusaka, Zambia.
[2]USDA-ARS, Sugarbeet and Bean Research Unit, 1066 Bogue St., East Lansing, MI 48824, USA.
[3]Dept. of Plant Sciences / MS1, Section of Crop & Ecosystem Sciences, University of California, 1 Shields Avenue, Davis, CA 95616-8780, USA.
[4]Zambia Agricultural Research Institute, Kasama, Zambia.

*Corresponding author
E-mail: kelvinkamfwa@gmail.com

Abstract

Anthracnose caused by *Colletotrichum lindemuthianum* is a major disease of common bean (*Phaseolus vulgaris*) worldwide. Yellow beans are a major market class of common bean especially in eastern and southern Africa. The objectives of this study were i) evaluate the yellow bean collection of 255 genotypes for resistance to races 5, 19, 39, 51, 81, 183, 1050 and 1105 of *C. lindemuthianum*., and ii) conduct genome-wide association analysis to identify genomic regions and candidate genes associated with resistance to *C. lindemuthianum*. The Yellow Bean Collection was genotyped with 72,866 SNPs using Genotyping by Sequencing, and genome-wide association analysis was conducted using Mixed Linear Model in TASSEL. Andean and Middle American genotypes with superior levels of resistance to the eight races ~~used in the current study~~ were identified. The genotype YBC278 was the only one among 255 genotypes that was highly resistant to all eight races. Resistance in the Yellow Bean Collection to the eight races used in the current study was controlled by major-effect loci on chromosomes Pv01, Pv03, Pv04, Pv05 and Pv07. The genomic region on Pv01, which overlapped with the Andean locus *Co-1* provided resistance to races 81, 1050 and 1105. Significant SNPs for resistance to race 39 were identified on Pv02. The genomic region on Pv04, which overlapped with known major-effect loci *Co-3*, *Co-15*, *Co-16*, *Co-y* and *Co-z*, provided resistance to races 5, 19, 51 and 183. Novel genomic regions for resistance to race 39 were identified on Pv05 and Pv07. Plant resistance genes (R genes) with NB-ARC and LRR domains, which occurred in clusters, were identified as positional candidate genes for genomic regions on Pv02 and Pv04.

Introduction

Common bean (*Phaseolus vulgaris*) is a source of food and nutritional security for millions of households in Latin America and Africa [1]. Beans play an important role in combating malnutrition because of its richness in protein, carbohydrates and minerals (especially iron and zinc) [2].

Beans have a rich diversity in traits such as seed color, seed size and seed shape [3]. These traits have been used to classify beans in different market classes. One of the market classes is the yellow bean, which is comprised of both Andean and Middle American genotypes. Within the yellow bean market class, there are several seed types classifications based on the shade of yellow color. These include the Manteca, Canary (Canario), Mayocoba, and Njano classes [4, 5]. Various yellow bean market classes are commercially important in Latin America, the Caribbean, and Africa [6, 7]. In Africa, yellow beans are popular in Angola, Kenya, Tanzania and Zambia [7]. In Zambia, the Manteca yellow landrace known as Lusaka is preferred over other bean market classes and fetches higher market prices [8]. In Tanzania, the Njano class is the most popular yellow seed type and it is estimated that about 32% of total bean production there is for yellow beans, and mainly the Njano type [9]. Yellow beans have become increasingly important bean corridors of both East and Southern Africa [10].

Consumers prefer yellow beans to other market classes because of their superiority in several end-use traits. Manteca yellow beans have shorter cooking time and higher iron bioavailability than other market classes [11-14]. Despite the consumer preference and

nutritional attributes of some yellow bean market classes have historically received less breeding efforts compared to other popular bean market classes.

Average yields for yellow bean are often lower than other bean classes. Several abiotic and biotic stresses contribute to low yields [15]. In some countries, continued use of yellow bean landraces with poor genetics and yield potential contribute to low yields. Diseases are a major contributing factor to the low yields of yellow beans. Anthracnose (ANTH) (caused by *Colletotrichum lindemuthianum*) is a major disease that affects yellow beans. Depending on varietal susceptibility and weather conditions (high humidity and moderate temperatures favors its spread), ANTH can cause yield losses of up to 100% [16]. ANTH is a seed borne disease and planting infected seed is a major mode of its transmission [17, 18]. This transmission mode of ANTH makes its control/or management challenging because of the widespread use of on-farm saved seed in Africa and Latin America. Though ANTH can effectively be controlled using fungicides, smallholder farmers who are the majority of bean growers cannot afford fungicides. Additionally, there are health and safety concerns involved in using fungicides. Development and use of resistant varieties is the most cost-effective and ecologically-sound management strategy for anthracnose.

ANTH resistance exists within the primary gene pool of common bean and several sources of resistance have been identified and used in breeding. Major-effect genes mainly control resistance to ANTH in common bean, though minor QTL have also been identified [19,20]. To date, 19 dominant major-effect ANTH resistance genes have been

identified in common bean. These genes are classified as either Andean or Middle American depending on whether they were identified in an Andean or Middle American genotype. Andean genes include *Co-1, Co-12, Co-13, Co-14, Co-15, Co-x, Co-w, Co-y*, and *Co-z* while Middle American genes include *Co-2, Co-3, Co-4, Co-5, Co-6, Co-11, Co-16, Co-17, Co-u,* and *Co-v* [21-24]. The genes *Co-1, Co-3, Co-4* and *Co-5* are multi-allelic. Some of these major genes are located in genomic regions that contain clusters of disease resistance genes that control resistance to multiple diseases [25, 26]. For example, *Co-1* is located in the genomic that also contain *Pgh-1,* which is a major gene for resistance to angular leaf spot. In addition to the major genes, QTL with smaller effect on ANTH resistance have been reported [27].

The major challenge in breeding for durable ANTH resistance is the broad genetic diversity of *Colletotrichum lindemuthianum*. To date, over 147 races of *C. lindemuthianum* have been reported worldwide [28]. *C. lindemuthianum* races have co-evolved with the two gene pools of common beans and are classified as either Andean of Middle American [21]. Andean races are virulent mostly on Andean beans while Middle American races are virulent on both Andean and Middle American beans. Because of the diversity of *C. lindemuthianum*, resistance often breaks down; therefore, there is need for continuous identification of new sources of resistance and understanding the genetic architecture of that resistance. Major genes for ANTH resistance follow gene-for-gene disease resistance concept. Therefore, some genes are effective against some races, but less effective against others. It is therefore important to identify genes that are effective against specific races so as to develop varieties with effective and durable resistance for a

given geographic location. Diversity panels are important genetic resources that can serve as sources of resistance and also for understanding the genetic architecture of traits such as *C. lindemuthianum*. The Yellow Bean Collection (YBC) is one of the diversity panels that have been assembled in common bean [4]. The YBC is comprised of landraces, varieties and elite lines of yellow beans, which are variable for several traits including anthracnose resistance. The YBC, therefore, is an excellent genetic resource for understanding the extent of ANTH resistance and its genetic architecture in the yellow bean market class.

QTL mapping using recombinant inbred lines and genome-wide association studies (GWAS) using diversity panels are two commonly used genetic approaches to understand the genetic architecture of traits. Both approaches have previously been used to identify genomic regions for resistance to variable races of *C. lindemuthianum* in common bean. One of the advantages of GWAS over QTL mapping is the higher mapping resolution that it offers, as GWAS takes advantage of historical recombination, leading to smaller linkage disequilibrium blocks. The enhanced mapping resolution of GWAS offers a better opportunity to precisely identify candidate genes for resistance to anthracnose. Additionally, GWAS using diversity panels provide an opportunity to identify minor effect QTL that QTL mapping populations using recombinant inbred lines cannot identify. Minor QTL are important sources of horizontal resistance, and if put in the same genetic background with major effect genes, which offers vertical resistance, they can confer durable resistance to multiple races of *C. lindemuthianum*. The objectives of this study were i) evaluate the yellow bean collection for resistance to eight races of *C.*

lindemuthianum, and ii) conduct genome-wide association analysis to identify genomic regions and candidate genes associated with resistance to eight races of *C. lindemuthianum*.

Materials and methods

Plant materials

In the current study the Yellow Bean Collection (YBC) of 255 genotypes consisting of landraces, breeding lines and varieties with variable shades of yellow seed colors from Africa, North America, South America, Europe, Central America, Caribbean, Middle East and East Asia was used [4]. These shades of yellow seed colors include Amarillo dark and light, beige, brown, Canary (Mexican), Manteca, Mayocoba, and green-yellow (Njano). The YBC has both Andean and Middle American genotypes.

Evaluation of the YBC for resistance to anthracnose

A total of eight races (5, 19, 39, 81, 183, 1050 and 1105) of *C. lindemuthianum* were used in the current study. These eight races were characterized from isolates collected from major bean-growing regions of Zambia using a set of 12 differential cultivars [29]. The eight races used in the current study offered a broad spectrum of virulence. Inoculation of the YBC followed a protocol described by Mungalu et al. 2020. This protocol involved planting the YBC (255 genotypes), anthracnose susceptible check (Kabulangeti) and anthracnose resistant check (G2333) on Styrofoam trays. A completely randomized design with three replications was used. Each replication had two seedlings

in a well. Therefore, a total of six seedlings per genotype were evaluated for resistance to each race. Inoculation was conducted when seedlings had developed fully expanded unifoliate leaves. Evaluation of the YBC was conducted separately for each of the eight races.

Inoculum preparation

Inoculum preparation was conducted using a protocol described in Mungalu et al. 2020. Each race was cultured on petri dishes on either potato dextrose agar (PDA) 39 g L^{-1} or modified Mathur's agar culture media made with dextrose (8 g L^{-1}), $MgSO_4 \cdot 7H_2O$ (2.5 g L^{-1}), KH_2PO_4 (2.7 g L^{-1}), neopeptone (2.4 g $L^{-1)}$, yeast extract (2.0 g L^{-1}), and agar (16 g L^{-1}). The petri dishes were incubated in the dark at 23-25 ºC for 7 to 10 days for sporulation. Sporulated plates where flooded with distilled water and allowed to settle for 20 minutes. Spores where then harvested by scrapping them off from the culture using a glass rod. The harvested spore suspension was then sieved through a double-layered cheesecloth into a clean beaker. The spore suspension concentration was then adjusted to 1.2 x 10^6 spores per ml using a hemocytometer as described in Mungalu et al. 2020. Tween 20 was added to the inoculum for adhesion. Seedlings were thoroughly sprayed with the inoculum on both surfaces of the primary leaves and the stem using a hand sprayer. After inoculation, plants were left to air-dry for a few minutes before they were placed in a high humidity chamber (>90% humidity) for 72 hrs at room temperature. After this incubation seedlings were removed from the humidity chamber and transferred to greenhouse benches where they stayed for 5 days for further anthracnose development. Anthracnose severity on seedlings was rated based on a 1-9 CIAT scale [34]. The score

range of 1-3 was considered as resistant and included plants with no visible symptoms or with few very small lesions, mostly on primary leaf veins, 4-6 was considered moderately resistant and included seedlings with small lesions on leaves and seedling stem, and 7-9 was considered susceptible and included dead seedlings and those with numerous small or enlarged lesions, with sunken cankers on leaves and seedling stem.

Severity score analyses

Statistical analyses on disease severity scores were conducted in SAS 9.3 [35]. Severity score data was first checked for normal distribution using PROC UNIVARIATE. Normality test results showed that severity scores for all races were normally distributed. Analysis of Variance was conducted using PROC MIXED following the mixed model:

$$Y_{ik}=\mu+\alpha_i+\gamma_k+E_{ik}$$

Where: Y_{ik} was anthracnose severity score, with genotype i, replication k; αi was the fixed variable effect of the genotype i; γ was the random variable effect of a replication; ε was the residual associated with replication k in genotype i. γk was a random variable, which was assumed to be normally distributed with mean = 0.

Genotypic data analyses

The YBC was genotyped with 72,866 Single Nucleotide Polymorphism (SNPs) markers using Genotyping by Sequencing (GBS). The DNA sequencing libraries were prepared using a single restriction enzyme ApeKI. The Genomics Core at Michigan State University sequenced the libraries using an Illumina standard HiSeq 4000 to generate 150

bp single-end reads. Additional details on genotyping can be found in Sadohara et al. 2022. The SNP data was used in population structure, kinship and SNP-disease severity analyses.

Population structure

Correction for population structure in a diversity panel such as the YBC is necessary to avoid false positives in GWAS. In the current study, the population structure in the YBC was determined using Principal Component Analysis (PCA) in TASSEL v.5.0 [36]. The first five principal components, which together accounted for 62.5% of variation in the YBC, were used as covariates in the GWAS analyses.

Kinship

Kinship (cryptic relatedness) among genotypes in a diversity panel such as the YBC can result on false positive. In the current study, kinship among genotypes was investigated and corrected for in GWAS using the Identical by Descent method in TASSEL.

Association analysis for anthracnose resistance

SNPs significantly associated with resistance to races 5, 19, 39, 51, 81, 183, 1050 and 1105 were identified using the following Mixed Linear Model [37]:

$$Y = G + P + K + \varepsilon$$

Where Y the phenotype of a genotype; X was the fixed effect of the SNP; P was the fixed effect of population structure (from PCA matrix from TASSEL); K was the random effect of relative kinship (from kinship matrix from TASSEL); ε was the error term, which was assumed to be normally distributed with mean = 0. MLM analysis was conducted in the software TASSEL v.5.0. Before use in MLM, SNP data was filtered for minor allele frequency (MAF=5%), which reduced the number of usable SNPs for MLM to 55,000. The Bonferroni-corrected *P*-value of 1.0 x 10^{-6} ($\alpha = 0.05$; 50,000 SNPs) was used as a threshold to determine the significance of the association between the SNP and severity score of a given race. The Manhattan plots for visualization of significant SNPs were made using a custom R script.

Identification of candidate genes

Candidate genes in genomic regions associated with resistance to the eight races of ANTH in the current study were identified from *Phaseolus vulgaris* v2.1 [38] in Phytozome using JBrowse following a previously described method [39]. Briefly, a gene was identified as a positional candidate gene based on two criteria: (i) it was within a genomic region of 400 kb of either upstream or downstream of the most significant SNP, and (ii) the functional role of a gene in disease resistance has been determined or proposed.

Results

Anthracnose severity score analysis

Highly significant (P<0.01) differences were observed among YBC genotypes in their reaction to races 5, 19, 39, 51, 81, 183, 1050 and 1105 of *C. lindemuthianum*. Severity scores for the YBC ranged from 1 to 9 for all eight races (Table 1).

~~**Table 1. Means of anthracnose severity score of G2333 (resistant check), Kabulangeti (susceptible check) and 255 lines of the YBC evaluated for resistance in the greenhouse at University of Zambia, Lusaka, Zambia.**~~

	**Checks**		**YBC**		
**ANTH race**	**G2333**	**Kabulangeti**	**Mean**	**Range**	**ANOVA**
**Race 5**	1.0	9.0	6.7±0.2	1.0-9.0	***
**Race 19**	1.0	9.0	6.3±0.21	1.0-9.0	***
**Race 39**	1.0	9.0	6.7±0.19	1.0-9.0	***
**Race 51**	1.0	9.0	5.2±0.21	1.0-9.0	***
**Race 81**	1.0	9.0	5.9±0.18	1.0-9.0	***
**Race 183**	1.0	9.0	6.4±0.2	1.0-9.0	***
**Race 1050**	1.0	9.0	6.5±0.19	1.0-9.0	***
**Race 1105**	1.0	9.0	5.7±0.21	1.0-9.0	***

*** = Signifiacant ($p < 0.001$)

The average severity score for the YBC to races 5, 19, 39, 51, 81, 183, 1050 and 1105 were 6.7, 6.3, 6.7, 5.2, 5.9, 6.4, 6.5 and 5.7, respectively (Table 1). The highest mean severity scores for the YBC were observed for Andean races 5 and 39. The frequency distribution of severity scores showed a bimodal distribution for all eight races (Fig 1).

Fig 1. Frequency distributions of severity scores for races 5, 19, 39, 51, 81, 183, 1050 and 1105 of *C. lindemuthianum* inoculated on the Yellow Bean Collection, resistant check (G2333) and susceptible check (Kabulangeti).

The resistant and susceptible checks reacted to all eight races as expected. The resistant (G2333) and susceptible (Kabulangeti) checks consistently scored 1 and 9, respectively, for all eight races (Table 1). Of the 255 YBC genotypes evaluated, five genotypes (YBC278, YBC130, YBC173, YBC192 and YBC267) showed superior resistance to most of the eight races (Table 2).

Table 2. Mean anthracnose severity scores for the five genotypes identified from the 255 Yellow Bean Collection genotypes as being highly resistant against races 5, 19, 39, 51, 81, 183, 1050 and 1105 of *Colletotrichum lindemuthianum* after artificial inoculation in the greenhouse at University of Zambia, Lusaka, Zambia.

YBC ID	ID	Country	Seed color	Genepool	Mean anthracnose scores							
					Race 5	Race 19	Race 39	Race 51	Race 81	Race 183	Race 1050	Race 1105
**YBC278**	SMC28	Uganda	Amarillo	Middle-American	1.0	1.0	1.0	1.0	1.0	1.0	1.0	1.0
**YBC130**	P1527538	Burundi	Green yellow	Andean	1.0	1.0	-	1.2	1.0	1.0	1.0	1.0
**YBC173**	G22501	Burundi	Green yellow	Andean	1.0	1.0	9.0	1.0	1.0	1.0	1.3	1.0
**YBC267**	DAB933	Uganda	Amarillo	Andean	3.3	1.0	1.0	1.0	1.3	3.5	-	1.0
**YBC192**	Roba-1	Uganda	Amarillo	Middle-American	1.0	1.3	-	1.0	6.8	1.0	1.7	1.0

The genotype YBC278 was ~~the only one that that was~~ highly resistant (severity score < 1) to all eight races. The severity scores for all 255 YBC genotypes for the eight races are presented in ~~S1 Table~~.

Genome-wide association analysis

SNPs significantly associated with resistance to races 5, 19, 39, 51, 81, 183, 1050 and 1105 were identified on chromosomes Pv01, Pv02, Pv04, Pv05 and Pv07 (Fig 2-9 , Table 3).

Fig 2. Manhattan plot showing SNPs on chromosome Pv01 significantly associated with resistance to race 81 of the Yellow Bean Collection genotypes evaluated in the greenhouse at University of Zambia, Lusaka, Zambia. The red solid horizontal line is the Bonferroni adjusted *P*-value (1.0E-06).

Fig 3. Manhattan plot showing SNPs on chromosome Pv01 significantly associated with resistance to race 1050 of the Yellow Bean Collection genotypes evaluated in the greenhouse at University of Zambia, Lusaka, Zambia. The red solid horizontal line is the Bonferroni adjusted *P*-value (1.0E-06).

Fig 4. Manhattan plot showing SNPs on chromosome Pv01 significantly associated with resistance to race 1105 of the Yellow Bean Collection genotypes evaluated in the greenhouse at University of Zambia, Lusaka, Zambia. The red solid horizontal line is the Bonferroni adjusted *P*-value (1.0E-06).

Fig 5. Manhattan plot showing SNPs on chromosome Pv02, Pv04, Pv05 and Pv07 significantly associated with resistance to Andean ANTH race 39 of the Yellow Bean Collection genotypes evaluated in the greenhouse at University of Zambia, Lusaka, Zambia. The red solid horizontal line is the Bonferroni adjusted *P*-value (1.0E-06).

Fig 6. Manhattan plot showing SNPs on chromosome Pv04 significantly associated with resistance to race 19, 51 and 183 of the Yellow Bean Collection genotypes evaluated in the greenhouse at University of Zambia, Lusaka, Zambia. The red solid horizontal line is the Bonferroni adjusted *P*-value (1.0E-06).

Fig 7. Manhattan plot showing SNPs on chromosome Pv04 significantly associated with resistance to race 51 of the Yellow Bean Collection genotypes evaluated in the greenhouse at University of Zambia, Lusaka, Zambia. The red solid horizontal line is the Bonferroni adjusted *P*-value (1.0E-06).

Fig 8. Manhattan plot showing SNPs on chromosome Pv04 significantly associated with resistance to race 183 of the Yellow Bean Collection genotypes evaluated in the greenhouse at University of Zambia, Lusaka, Zambia. The red solid horizontal line is the Bonferroni adjusted *P*-value (1.0E-06).

Fig 9. Manhattan plot showing SNPs on chromosome Pv04 significantly associated with resistance to Andean ANTH race 5 in the Yellow Bean Collection evaluated in

the greenhouse at University of Zambia, Lusaka, Zambia. The red solid horizontal line is the Bonferroni adjusted *P*-value (1.0E-06).

~~**Table 3. SNPs markers significantly associated with resistance to races 5, 19, 39, 51, 81, 183, 1050 and 1105 of *Colletotrichum lindemuthianum* identified using the Yellow Bean Collection evaluated in the greenhouse at University of Zambia, Lusaka, Zambia.**~~

Trait	Chr	SNP	Position (bp)	*P*-value	R^2 (%)
**Race 5**	Pv04	Chr04_40179029	4,0179,029	1.5E-06	12.6
**Race 19**	Pv04	Chr04_2140558	2,140,558	1.1E-11	24.7
**Race 39**	Pv02	Chr02_49318523	49,318,523	7.4E-10	30.1
**Race 39**	Pv05	Chr05_15211384	15,211,384	1.19E-07	13.3
**Race 39**	Pv07	Chr07_27213163	27,213,163	7.15E-07	14.2
**Race 51**	Pv04	Chr04_2140558	2,140,558	7.6E-10	18.8
**Race 81**	Pv01	Chr01_49584097	49,584,097	6.2E-08	41.2
**Race 183**	Pv04	Chr04_1067693	1,067,693	3.4E-11	22.5
**Race 1050**	Pv01	Chr01_49583965	49,583,965	1.9E-12	27.5
**Race 1105**	Pv01	Chr01_49584097	49,584,097	1.1E-10	21.4

Chr = Chromosome; bp = base pairs; *P*-value = level of significance; E = exponential; R^2 = proportion of variation of anthracnose resistance explained by the significant SNP

Chromosome Pv01

Several SNPs in the genomic region spanning 49,151,104 bp - 49,584,097 bp on chromosome Pv01 were significantly associated with resistance to races 81, 1050 and 1105 (Fig 2 - 4). The most significant SNP (Chr01_49584097) in this genomic region

explained 41.2%, 27.5% and 21.4% of the YBC variation in ANTH severity caused by races 81, 1050 and 1105, respectively (Table 3).

Chromosome Pv02

Significant SNPs for resistance to the Andean ANTH race 39 were identified on Pv02 in a genomic region spanning from 49,155,240 bp - 49,416,064 bp (Fig 5). The most significant SNP (Chr02_49318523) in this genomic region explained 30.1% of the YBC variation in ANTH severity caused by race 39 (Table 3).

Chromosome Pv04

Significant SNPs for races 19, 51 and 183 were identified in separate genomic regions of Pv04 (Fig 6 - 8). The first genomic region was at the beginning (1,067,693 bp - 2,140,558 bp) of Pv04. Several SNPs in this genomic region were associated with resistance to races 19, 51 and 183. The R^2 for the most significant SNP (Chr04_2140558, Chr04_2140558 and Chr04_1067693) for races 19, 51 and 183 were 24.7%, 18.8% and 22.5%, respectively. The second genomic region identified on Pv04 was at 40,179,029 bp. This region was significantly associated with resistance to race 5 and the most significant SNP at this region explained 12.6% ~~of the~~ variation in the YBC for ANTH severity caused by race 5.

Chromosome Pv05 and Pv07

SNPs significantly associated with resistance to race 39 were identified on Pv05 and Pv07 (Figu 9). The most significant SNPs on Pv05 (Chr05_15211384; 15,211,384 bp)

and Pv07 (Chr07_27213163; 27,213,163 bp) explained 13.3% and 14.2% of the variation in severity scores for race 39 (Table 3).

Discussion

Anthracnose caused by *C. lindemuthianum* is a major disease of common bean. The productivity of yellow beans, a major market class in several African countries, is constrained by anthracnose. In the current study, we evaluated the YBC for its reaction to races 5, 19, 39, 51, 81, 183, 1050 and 1105 of *C. lindemuthianum*, and conducted GWAS to identify genomic regions and positional candidate genes conferring resistance to these eight races.

The frequency distribution graphs for severity for all eight races showed a bimodal pattern, suggesting that major-effect genes were the basis of ANTH resistance, which is consistent with previous description of ANTH resistance in common bean as being controlled by major-effect genes. Identification of sources of resistance to anthracnose within the yellow bean market class would necessitate progress in the genetic enhancement of yellow beans for resistance to anthracnose. Such identification is necessary because of the challenges associated with poor recovery of yellow seed color and seed size in progenies from crosses of yellow bean with other colors. The current study has confirmed the existence of adequate levels of ANTH resistance to both Andean and Middle-American races of *C. lindemuthianum* in the yellow bean market class. In this study the genotype YBC278 was identified as having superior resistance to ANTH. YBC278 was the only genotype in the YBC that was highly resistant (severity score of

less than 1) to all the eight races used in the current study. This reaction was only similar to that of the resistant check G2333, a Middle-American genotype widely reported to be highly resistant to both Andean and Middle-American races of *C. lindemuthianum*. YBC278, also known as SMC28, is a Middle American bean of race Mesoamerica from CIAT-Uganda. It has an Amarillo (dark yellow) seed type and a 100-seed weight of 27 grams. The identification of YBC278 as highly resistant to both Middle American and Andean genotypes is important, as it will serve as a source of superior resistance for developing yellow bean varieties with durable resistance to anthracnose. Genotypes YBC130 and YBC267 are the other two genotypes that showed superior levels of resistance to ANTH. YBC130 is an Andean landrace from Burundi with a green-yellow seedcoat and a 100-seed weight of approximately 43 grams. It was included in the Andean Diversity Panel as ADP0468 and is listed as PI 527538 in the USDA-NPGS gene bank. YBC267, also known as DAB 933, is an Andean bean from CIAT-Uganda of the Amarillo (dark) market category, with a 100-seed weight of 30 grams. These accessions could be used as alternative sources of ANTH resistance for yellow bean breeding.

The genetic basis of the observed resistance in the YBC to eight races of *C. lindemuthianum* was explored using genome-wide association analysis. Genomic regions associated with resistance to the eight races used in the current study were identified on chromosomes Pv01, Pv02, Pv04, Pv05 and Pv07.

Several SNPs within a 528 kbp region (49,151,104-49,679,631 bp) on Pv01 were significantly associated with resistance to race 81, and the highly virulent races 1050 and

1105. This is a major-effect QTL and it appears to be the major locus *Co-1*, which confers resistance mainly to Middle American races of *C. lindemuthianum*. The SNPs most significantly associated with resistance to races 81, 1050, and 1105 were found at positions 49,584,097, 49,583,965, and 49,584,097 of Pv01, respectively. In each case, the ~~most~~ SNP most significantly associated with ANTH resistance is in the gene model Phvul.001G243800, a protein-kinase recently proposed to underlie *Co-1* [40,41]. This is a powerful validation of the role of this gene, as well as the experimental methods employed in this study. Previous studies have shown existence of both Andean and Middle American races in East and Southern African countries including Zambia, which underscores the importance of having *Co-1* gene in Andean genotypes such as the yellow beans.

The genomic region 49,318,523 bp on Pv02 contained SNPs that were significantly associated with resistance to races 19 and 39. The identification of YBC genotypes resistant to races 19 and 39, and their associated resistance genomic regions has breeding significance to Zambia and other bean producing countries for two reasons. First, race 19 is the most prevalent race in the bean-producing regions of Zambia. Second, race 39 is a highly virulent Andean race [27] and it is important that yellow bean genotypes with superior resistance to race 39 are identified to serve as source of resistance for genetic improvement of yellow beans in countries where it is a major market class. The association between race 39 and the genomic region on Pv02 (49,318,523 bp) has previously been reported [27]. Eight plant disease resistance genes (R genes) including Phvul.002G323100, Phvul.002G323200, Phvul.002G323300, Phvul.002G323400,

Phvul.002G323404, Phvul.002G323704, Phvul.002G323708, and Phvul.002G323712 with NB-ARC and LRR domains were identified as positional candidate genes for resistance to race 39 identified on Pv02. R genes encode receptors that play a key role in the recognition of avirulence (Avr) products expressed by the pathogen during infection, which triggers an immune response and is the basis for gene-for-gene or race-specific resistance for ANTH observed in common bean.

The genomic region on 1.1 Mbp – 2.1 Mbp on Pv04 conferred resistance to races 19, 51 and 183. This genomic region overlaps with major-effect loci *Co-3*, *Co-15*, *Co-16*, *Co-y* and *Co-z*. The relatively large number of races whose resistance was controlled by this genomic is indicative of its significant role in the YBC as a source of broad-spectrum resistance to multiple races of *C. lindemuthianum*. The genomic region 1.1 Mbp – 2.1 Mbp identified in the current study ~~as~~ providing resistance to races 19, 51 and 183 was previously reported to also provide resistance to races 7, 19, 49, 55, 109, 530, 566 and 1331 [27, 30], which underscores the importance of this genomic region for resistance to multiple races of anthracnose. Five disease resistance (R) genes including Phvul.004G015600, Phvul.004G015800, Phvul.004G015900, Phvul.004G016000 and Phvul.004G016532 with the NB-ARC and LRR domains were identified as candidate genes for resistance to races 19, 51 and 183 at the 1.1 Mbp - 2.1 Mbp genomic region on Pv04. These R genes occurred in a cluster and it is plausible that some of them provide race-specific resistance because this genomic region provided resistance to a relatively large number of races. Given that *Co-3* has previously been reported as multi-allelic it is ~~plausible~~ that resistance to races 19, 51 and 183 reported in the current study may be

conditioned by different alleles. In addition to overlapping with *Co-3*, the genomic region on Pv04 identified in the current study also overlaps with the telomere cluster of resistance genes for rust (*Ur-5* and *Ur-14*) [42], angular leaf spot (*Phg-3*) [42], powdery mildew [43] and halo blight QTL (HB4.2) [44], which are major diseases of beans in Africa. Given the convergence of resistance to several races of ANTH and other major diseases at the identified genomic region in the current study, it is a worthwhile target for Marker-assisted selection to develop common bean varieties with durable resistance to ANTH and also multiple resistances to ANTH, rust and angular leaf spot. The current study has identified another genomic region on Pv04 ~~to~~ control resistance to ANTH. This genomic region located at the distal end on Pv04 (40.2 Mbp) is a major locus (R^2=12.6%) and the basis for resistance in the YBC to race 5 (Figure 5). This is the first report of ANTH resistance to race 5 at this genomic region.

Significant SNPs for resistance to race 39 were identified on Pv05 (15.2 Mbp) and Pv07 (27.2 Mbp). Given the large R^2 for the most significant SNPs on Pv05 (13.1%) and PV07 (15.5%) the two QTL had major effect on resistance to race 39 in the YBC. This is the first report of resistance for race 39 on Pv05 and Pv07. Banoo et al. (2020) identified significant SNPs for resistance to races 87 and 73 on Pv05 and Pv07, but in a genomic region different from the one identified for race 39 in the current study. Resistance to race 39 has previously been reported on other chromosomes but not in the genomic regions on Pv05 and Pv05 identified in the current study. The locus *Co-2* on Pv11 was previously identified using a population of RILs as the source of resistance to race 39 [26]. A previous GWAS conducted using the ADP reported resistance to race 39 only on

chromosome Pv04 [27], the same region we have also identified in the current study. Our study has not only identified a previously known genomic region on Pv04 as source of resistance to race 39, but has also identified novel major QTL on Pv05 and Pv07 as additional genomic regions for resistance to race 39.

Conclusion

Three YBC accessions with superior resistance to races 5, 19, 51, 81, 183, 1050 and 1105 were identified. The accession YBC278 was notable because it is the only one in the YBC that showed strong resistance to all eight races of *C. lindemuthianum* used in the current study.

The genetic architecture for resistance to eight races of *C. lindemuthianum* in the YBC has been determined. Major-effect loci on Pv01 (*Co-1*), Pv02 and Pv04 (which overlaps with *Co-3*, *Co-13*, *Co-15*, *Co-y* and *Co-z*) controlled most of the observed resistance to eight races observed in the YBC. In addition to the identified major-effect loci genomic regions on Pv01, Pv02 and Pv04, novel genomic regions on Pv04 (40.2 Mbp), Pv05 (15.2 Mbp) and Pv07 (27.2 Mbp) were significantly associated with the Andean races 5 and 39. Clusters of R genes with NB-ARC and LRR domains on Pv02 and Pv04 were identified as candidate genes.

Acknowledgement

We thank the University of Zambia Bean Breeding Program team for their assistance in conducting inoculations. We also thank Sadohara of Dr. Karen Cichy's for DNA collection.

References

1. Uebersax MA, Cichy KA, Gomez FE, Porch TG, Heitholt J, Osorno JM, et al. Dry beans (*Phaseolus vulgaris* L.) as a vital component of sustainable agriculture and food security. A review. Legume Science. 2022; e155.
2. Haas J, Luna S, Lung'aho M, Wenger M, Murray-Kolb L, Beebe S, et al. Consuming iron bio-fortified beans increases iron status in Rwandan women after 128 days in a randomized controlled feeding trial. J Nutr 2016; 146: 1586-1592
3. Singh SP. Broadening the genetic base of common bean cultivars: a review. Crop Sci. 2001; 41(6): 1659-1675.
4. Sadohara R, Izquierdo P, Couto Alves F, Porch T, Beaver J, Urrea CA, et al. The *Phaseolus vulgaris* L. Yellow Bean Collection: genetic diversity and characterization for cooking time. Genet Resour Crop Evol. 2022; 69(4): 1627-1648
5. Sadohara R, Long Y, Izquierdo P, Urrea CA, Morris D, Cichy K. Seed coat color genetics and genotype x environment effects in yellow beans via machine-learning and genome-wide association. Plant Genome. 2021; 15(1): e20173
6. Voysest O. Yellow beans in Latin America. Ann Rep Bean Improv Coop. 2021; xii–xviii.

7. Buruchara R, Chirwa R, Sperling L, Mukankusi C, Rubyogo JC, Mutonhi R, et al. Development and delivery of bean varieties in Africa: the Pan-Africa Bean Research Alliance (PABRA) model. Afr Crop Sci J. 2011; 19(4): 227-245.
8. Sichilima T, Mapemba L, Tembo, G. Drivers of dry common beans trade in Lusaka, Zambia: A trader's perspective. Sustain Agric Res. 2016; 5(2): 15-26.
9. Sperling L, Birachi E, Kalemera S, Mutua M, Templer N, Mukankusi C, et al. The informal seed business: focus on yellow bean in Tanzania. Sustainability. 2021; 13(16): 8897
10. Farrow A, Muthoni-Andriatsitohaina R, editors. Atlas of common bean production in Africa 2nd ed. Nairobi, Kenya: 2022.
11. Wiesinger JA, Cichy KA, Tako E, Glahn RP. The fast cooking and enhanced iron bioavailability properties of the Manteca yellow bean (*Phaseolus vulgaris* L.). Nutrients. 2018; 10(11): 1609.
12. Hart JJ, Tako E, Wiesinger J, Glahn RP. Polyphenolic profiles of yellow bean seed coats and their relationship with iron bioavailability. Agric Food Chem. 2019; 68(3): 769-778.
13. Cichy KA, Wiesinger JA, Mendoza FA. Genetic diversity and genome-wide association analysis of cooking time in dry bean (*Phaseolus vulgaris* L.). Theor Appl Genet. 2015; 128(8): 1555-1567.
14. Mishili FJ, Temu A, Fulton J, Lowenberg-DeBoer J. Consumer preferences as drivers of the common bean trade in Tanzania: A marketing perspective. J Int Food Agribus Mark. 2011; 23(2): 110-127.

15. Parker TA, Acosta Gallegos J, Beaver J, Brick M, Brown JK, et al. Genetic Resources and Breeding Priorities in Phaseolus Beans: Vulnerability, Resilience, and Future Challenges. In: Plant Breeding Reviews. 2023; 46: 289.

16. Pastor-Corrales MA, Tu JC. Anthracnose. In: H.F. Schwartz, and M.A. Pastor-Corrales, editors. Bean production problems in the tropics. CIAT, Colombia; 1989. p 77–104

17. Schwartz HF, Pastor-Corrales MA. Anthracnose. In: Schwartz HF, Steadman JR, Hall R, and Forster RL, editors. Compendium of Bean Diseases. St. Paul Minnesota: APS Press; 2005. p. 25–27.

18. Ferreira JJ, Campa A, Kelly JD. Organization of genes conferring resistance to anthracnose in common bean. In: Varshney RK, Tuberosa R, editors. Translational Genomic for Crop Breeding, Volume I: Biotic Stress. Chichester: John Wiley & Sons; 2013. p. 151–182

19. Nabi A, Lateef I, Nisa Q, Banoo A, Rasool RS, Shah MD, et al. Phaseolus vulgaris−Colletotrichum lindemuthianum pathosystem in the post−genomic era: an update. Curr. Microbiol. 2022; 79: 36.

20. Banoo A, Nabi A, Rasool RS, Shah MD, Ahmad M, Sofi PA, Itoo H, Sharma PN, Padder BA. North-western Himalayan common beans: population structure and mapping of quantitative anthracnose resistance through genome wide association study. Front Plant Sci. 2020; 11: 571618.

21. Geffroy V, Sicard D, de Oliveira JC, Sévignac M, Cohen S, Gepts P, et al. Identification of an ancestral resistance gene cluster involved in the coevolution

process between *Phaseolus vulgaris* and its fungal pathogen *Colletotrichum lindemuthianum*. Mol Plant Microbe Interact. 1999; 12(9): 774-784.

22. Gonçalves-Vidigal MC, Lacanallo GF, Vidigal PS. A new Andean gene conferring resistance to anthracnose in common bean (*Phaseolus vulgaris* L.) cultivar Jalo Vermelho. Plant Breed. 2008; 127: 592–596.
23. Gonçalves-Vidigal MC, Vidigal Filho PS, Medeiros AF, Pastor-Corrales MA. Common bean landrace Jalo Listras Pretas is the source of a new Andean anthracnose resistance gene. Crop Sci. 2009; 49: 133–138.
24. Gonçalves-Vidigal MC, Meirelles AC, Poletine JP, Sousa LL, Cruz AS, Nunes MP, et al. Genetic analysis of anthracnose resistance in ‘Pitanga’ dry bean cultivar. Plant Breed. 2012; 131: 423–429.
25. Oblessuc PR, Francisco C, Melotto M. The Co-4 locus on chromosome Pv08 contains a unique cluster of 18 COK-4 genes and is regulated by immune response in common bean. Theor Appl Genet. 2015; 128(6): 1193-1208.
26. Campa A, Trabanco N, Ferreira JJ. Identification of clusters that condition resistance to anthracnose in the common bean differential cultivars AB136 and MDRK. Phytopathology. 2017; 107(12): 1515-1521.
27. Zuiderveen GH, Padder BA, Kamfwa K, Song Q, Kelly JD. Genome-wide association study of anthracnose resistance in Andean beans. PLoS ONE. 2016; 11: e0156391.
28. Padder BA, Sharma PN, Awale HE, Kelly JD. *Colletotrichum lindemuthianum*, the causal agent of bean anthracnose. Plant Pathol. 2017; 317-330
29. Pastor-Corrales MA. Estandarización de variedades diferenciales y de designación de razas de *Colletotrichum lindemuthianum*. Phytopathology. 1991; 81(6): 694.

30. Mungalu H, Sansala M, Hamabwe S, Mukuma C, Gepts P, Kelly JD, et al. Identification of race-specific quantitative trait loci for resistance to *Colletotrichum lindemuthianum* in an Andean population of common bean. Crop Sci. 2020; 60(6): 2843-2856.

31. Campa A, Rodríguez-Suárez C, Giraldez R, Ferreira JJ. Genetic analysis of the response to eleven Colletotrichum lindemuthianum races in a RIL population of common bean (Phaseolus vulgaris L.). BMC Plant Biology. 2014; 14:115.

32. González AM, Yuste-Lisbona FJ, Rodiño AP, De Ron AM, Capel C, García-Alcázar M, Santalla M. Uncovering the genetic architecture of Colletotrichum lindemuthianum resistance through QTL mapping and epistatic interaction analysis in common bean. Front Plant Sci. 2015; 6: 141.

33. Murube E, Campa A, Ferreira JJ. Integrating genetic and physical positions of the anthracnose resistance genes described in bean chromosomes Pv01 and Pv04. PLoS ONE. 2019; 14: e0212298.

34. Balardin RS, Jarosz AM, Kelly JD. Virulence and molecular diversity in *Colletotrichum lindemuthianum* from South, Central, and North America. Phytopathology. 1997; 87(12): 1184-1191

35. SAS Institute. SAS version 9.3. SAS Institute Inc., Cary, NC. 2011.

36. Bradbury PJ, Zhang Z, Kroon DE, Casstevens TM, Ramdoss Y, Buckler ES. TASSEL: software for association mapping of complex traits in diverse samples. Bioinformatics. 2007; 23(19): 2633-2635.

37. Zhang Z, Ersoz E, Lai CQ, Todhunter RJ, Tiwari HK, Gore MA, et al. Mixed linear model approach adapted for genome-wide association studies. Nat Genet. 2010; 42(4): 355-360.

38. Schmutz J, McClean PE, Mamidi S, Wu GA, Cannon SB, Grimwood J, et al. A reference genome for common bean and genome-wide analysis of dual domestications. Nat Genet. 2014; 46(7): 707-713

39. Kamfwa K, Cichy KA, Kelly JD. Genome-wide association analysis of symbiotic nitrogen fixation in common bean. Theor Appl Genet. 2015; 128(10): 1999-2017.

40. Mahiya-Farooq, Padder BA, Bhat NN, Shah MD, Shikari AB, Awale HE, et al. Temporal expression of candidate genes at the Co-1 locus and their interaction with other defense related genes in common bean. Physiol Mol Plant Pathol. 2019; 108: 101424

41. Lima LR, Gonçalves-Vidigal MC, Vaz Bisneta M, Valentini G, Vidigal Filho PS, Martins VD, de Souza TL. Genetic fine-mapping of anthracnose disease-resistance allele Co-14 present in the Andean common bean cultivar AND 277. Crop Science. 2023;63(2):750-63.

42. Valentini G, Goncalves-Vidigal M, Cregan P, Song Q, Pastor-Corrales MA. High-resolution mapping reveals linkage between genes in common bean cultivar Ouro Negro conferring resistance to the rust, anthracnose, and angular leaf spot diseases. Theor Appl Genet. 2017; 130: 1705-1722.

43. Binagwa PH, Traore SM, Egnin M, Bernard GC, Ritte I, Mortley D, et al. Genome-Wide Identification of Powdery Mildew Resistance in Common Bean (*Phaseolus vulgaris* L.). Front Genet. 2021; 12: 673069.

44. Tock AJ, Fourie D, Walley PG, Holub EB, Soler A, Cichy KA, et al. Genome-wide linkage and association mapping of halo blight resistance in common bean to race 6 of the globally important bacterial pathogen. Front Plant Sci. 2017; 7(8): 1170.

Supporting information

S1 Table. Severity scores for races 5, 19, 39, 51, 81, 183, 1050 and 1105 of *C. lindemuthianum* for the 255 Yellow Bean Collection genotypes evaluated in the greenhouse at University of Zambia, Lusaka, Zambia

Figure

Click here to access/download;Figure;Fig 9.tiff

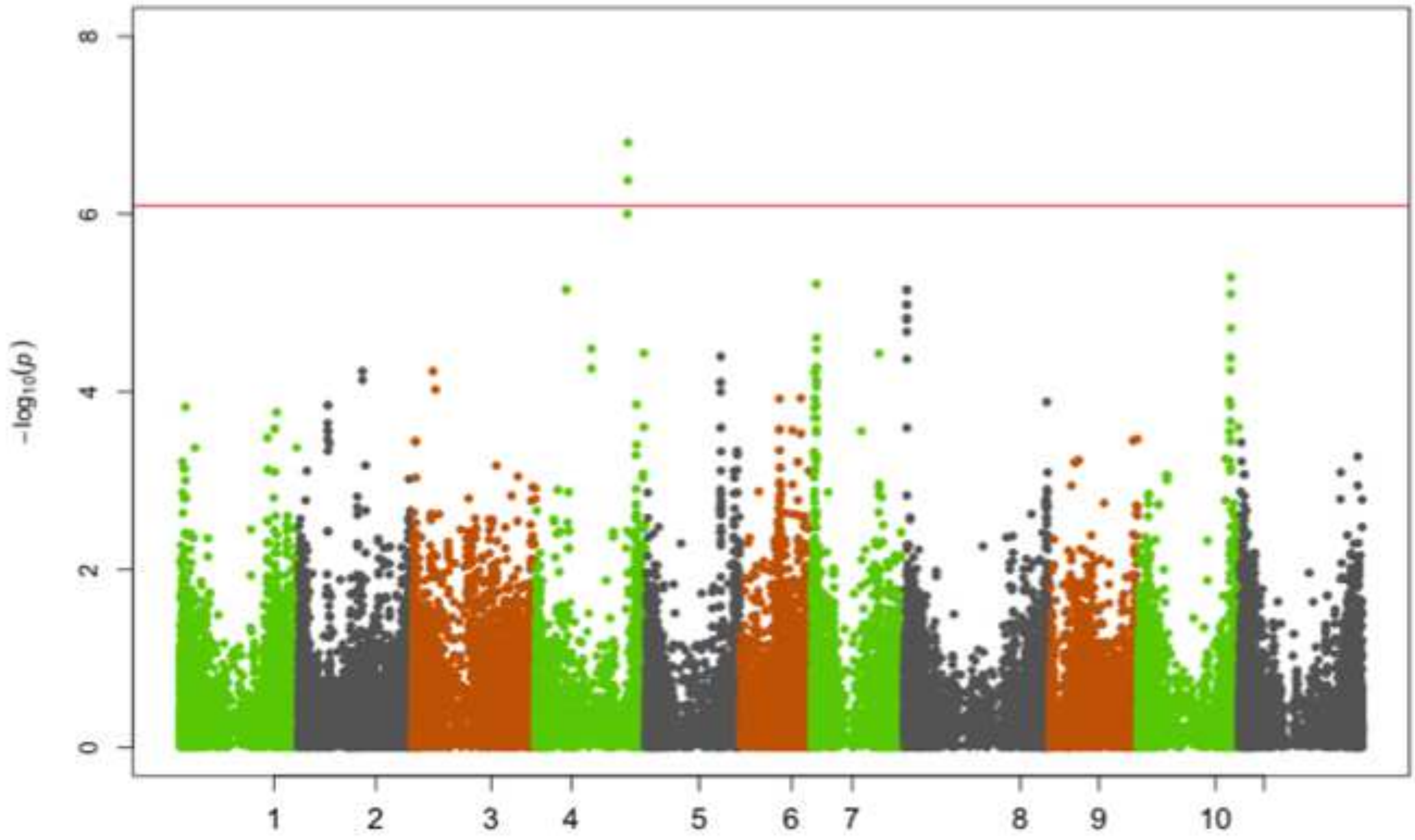

Figure

Click here to access/download;Figure;Fig 8.tiff

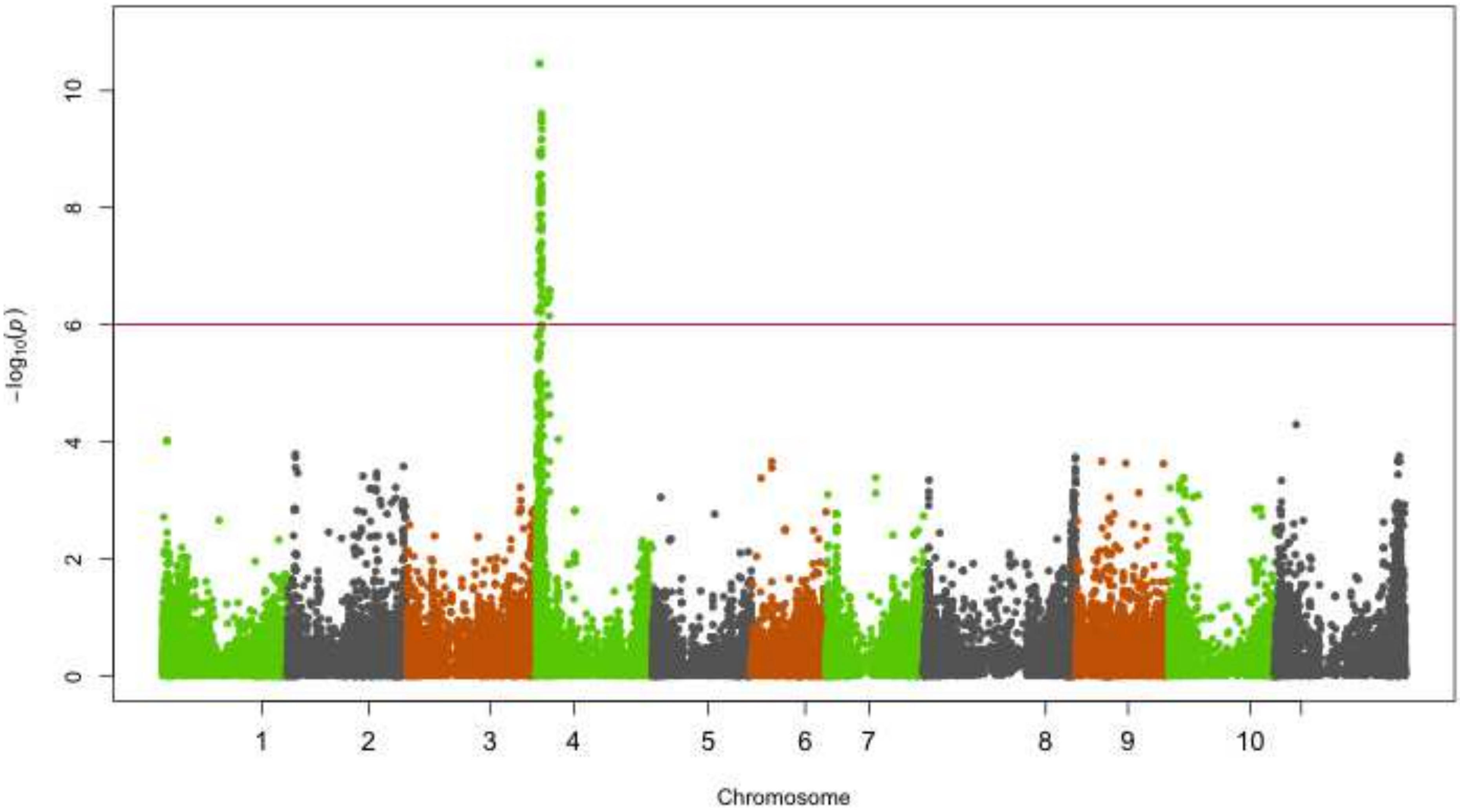

Figure

Click here to access/download;Figure;Fig 7.tiff

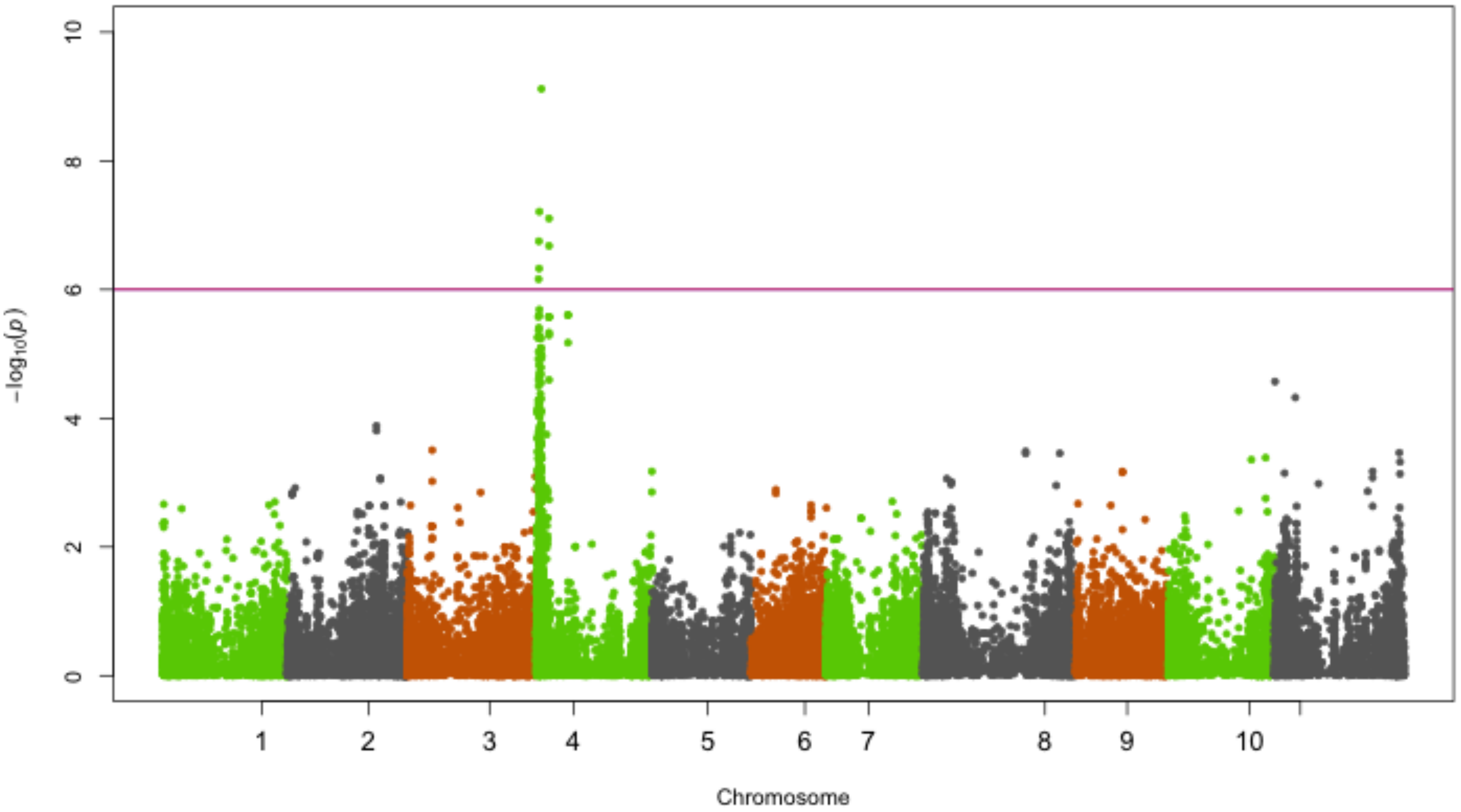

Click here to access/download;Figure;Fig 6.tiff

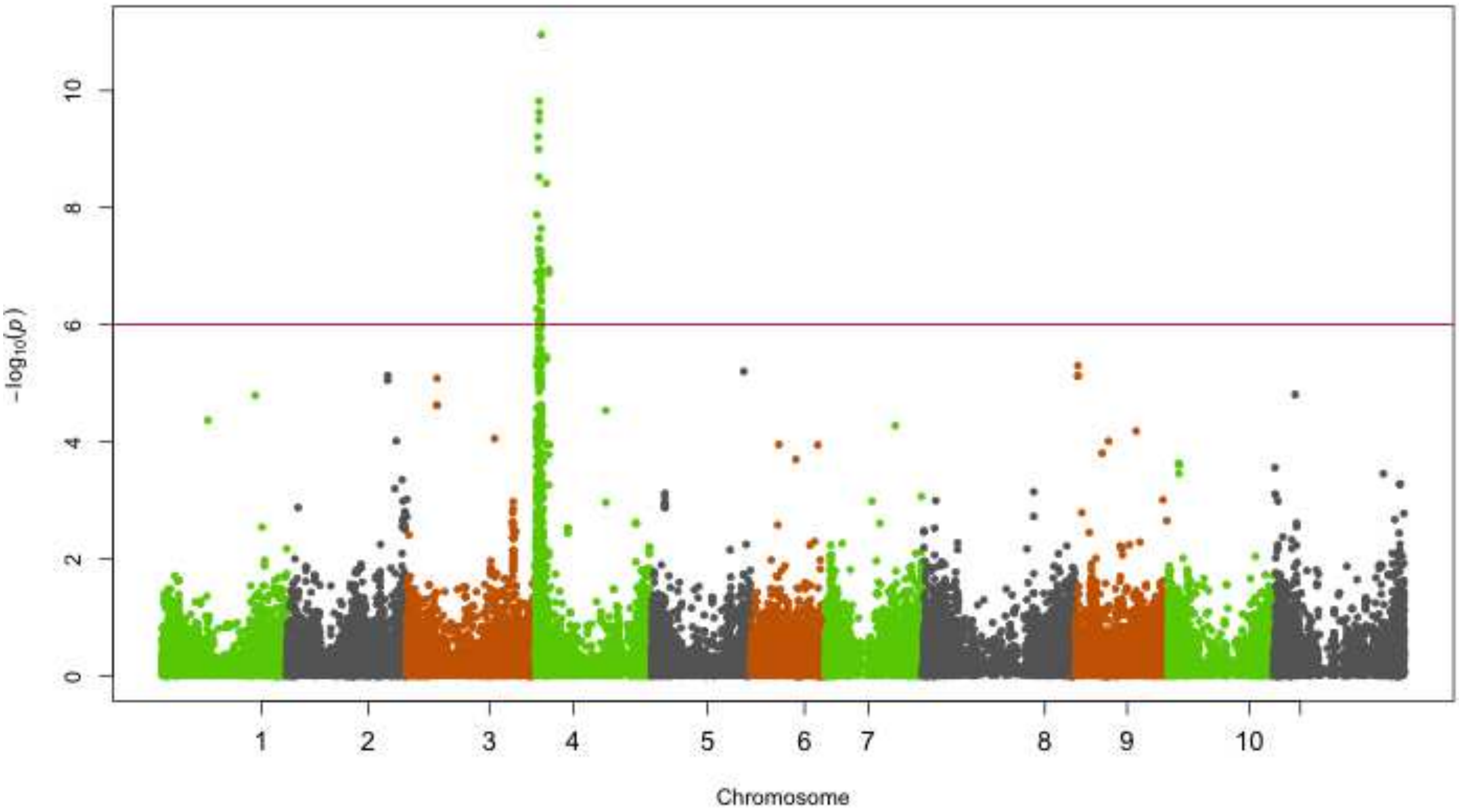

Click here to access/download;Figure;Fig 5.tiff

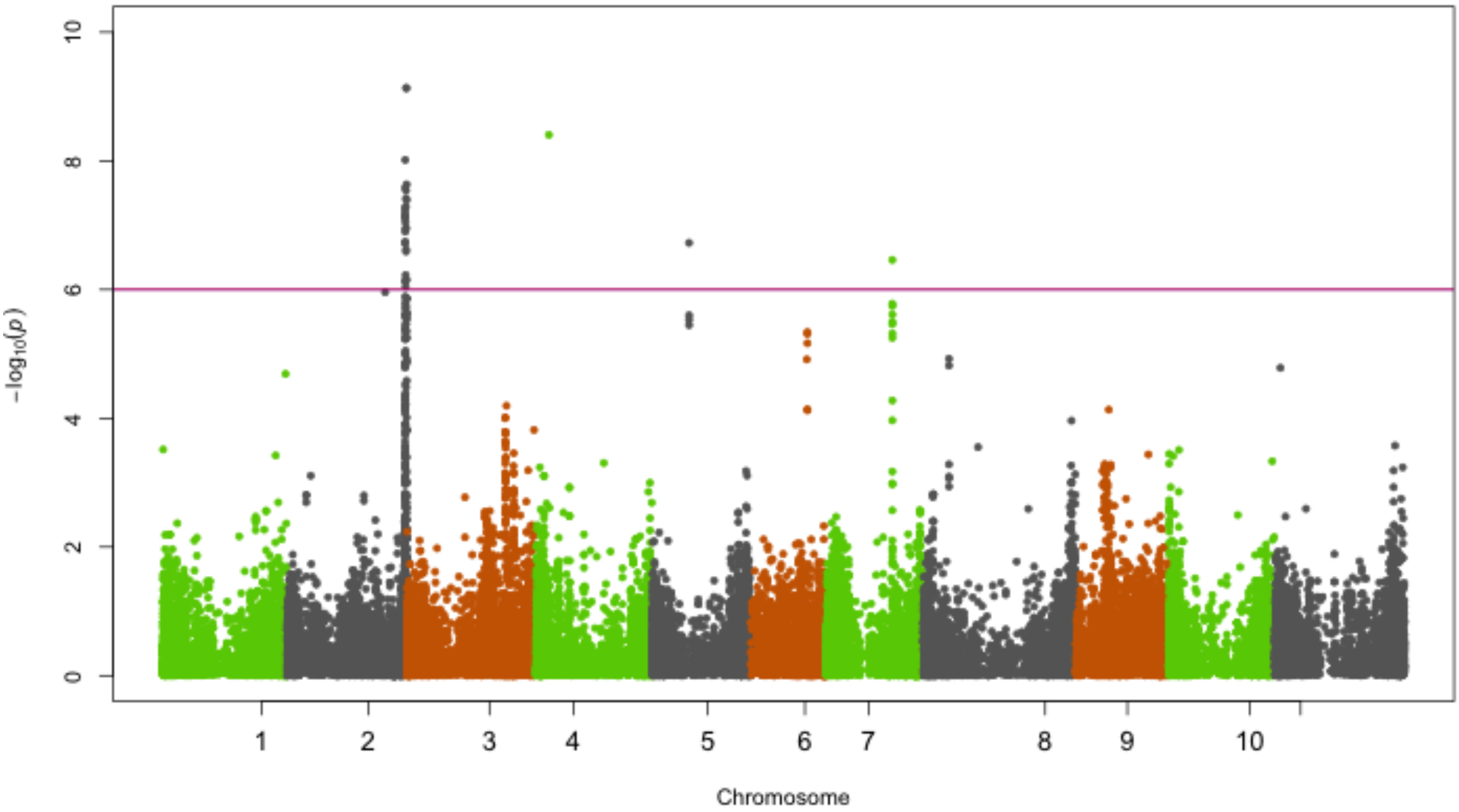

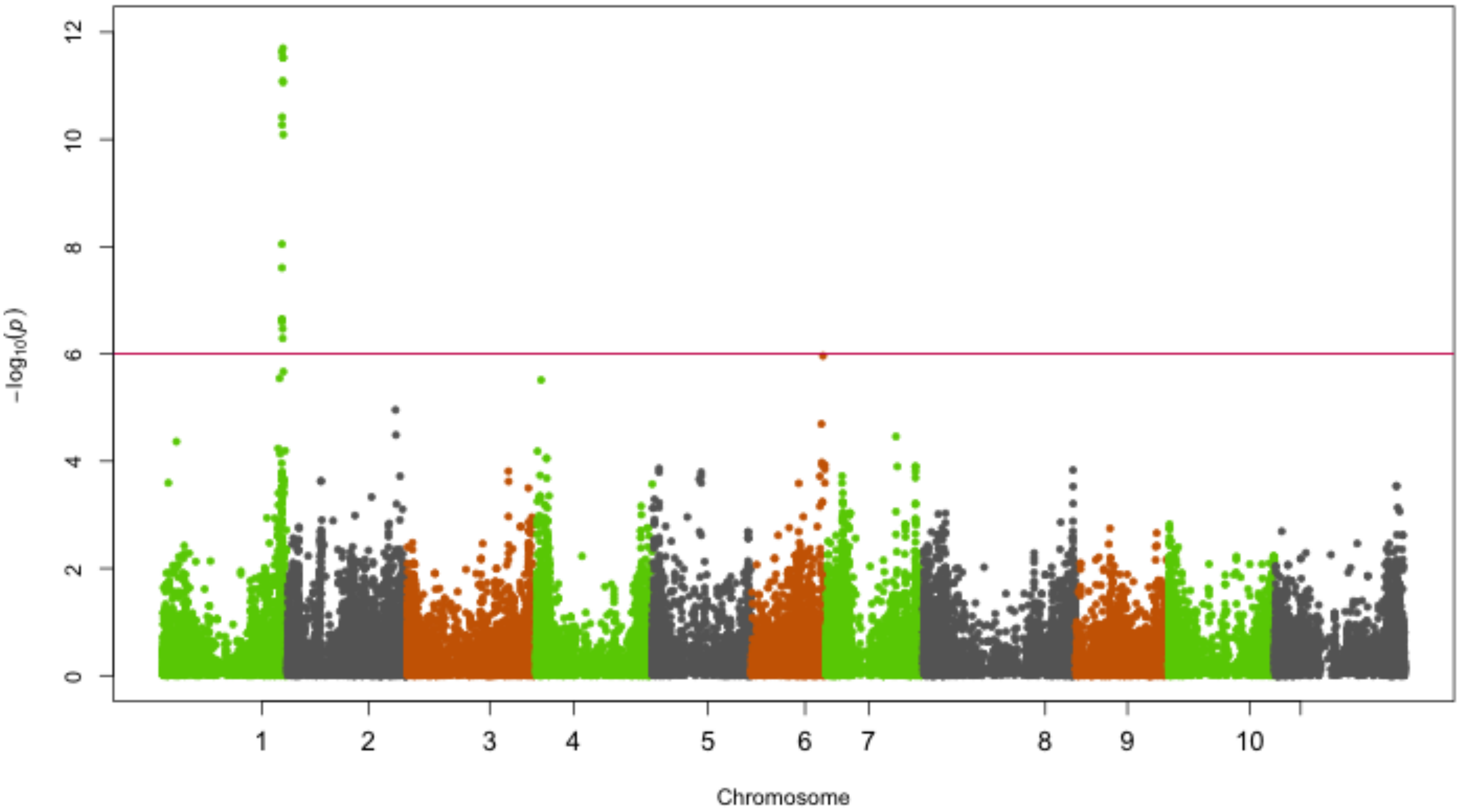
12
10
8
6
4
2
0
$-\log_{10}(p)$
1
2
3
4
5
6
7
8
9
10
Chromosome

Figure

Click here to access/download;Figure;Fig 4.tiff

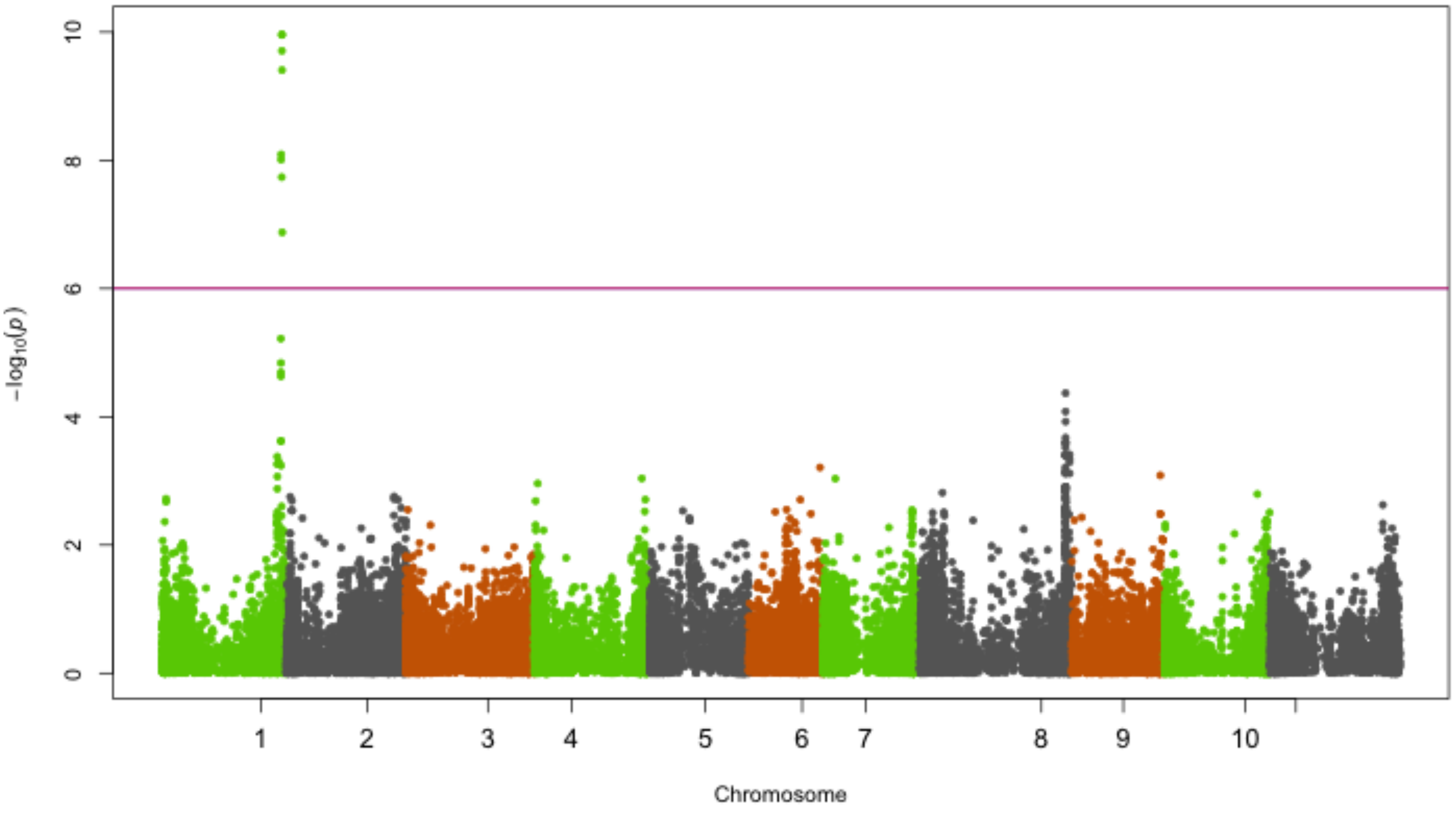

Figure

Click here to access/download;Figure;Fig 2.tiff

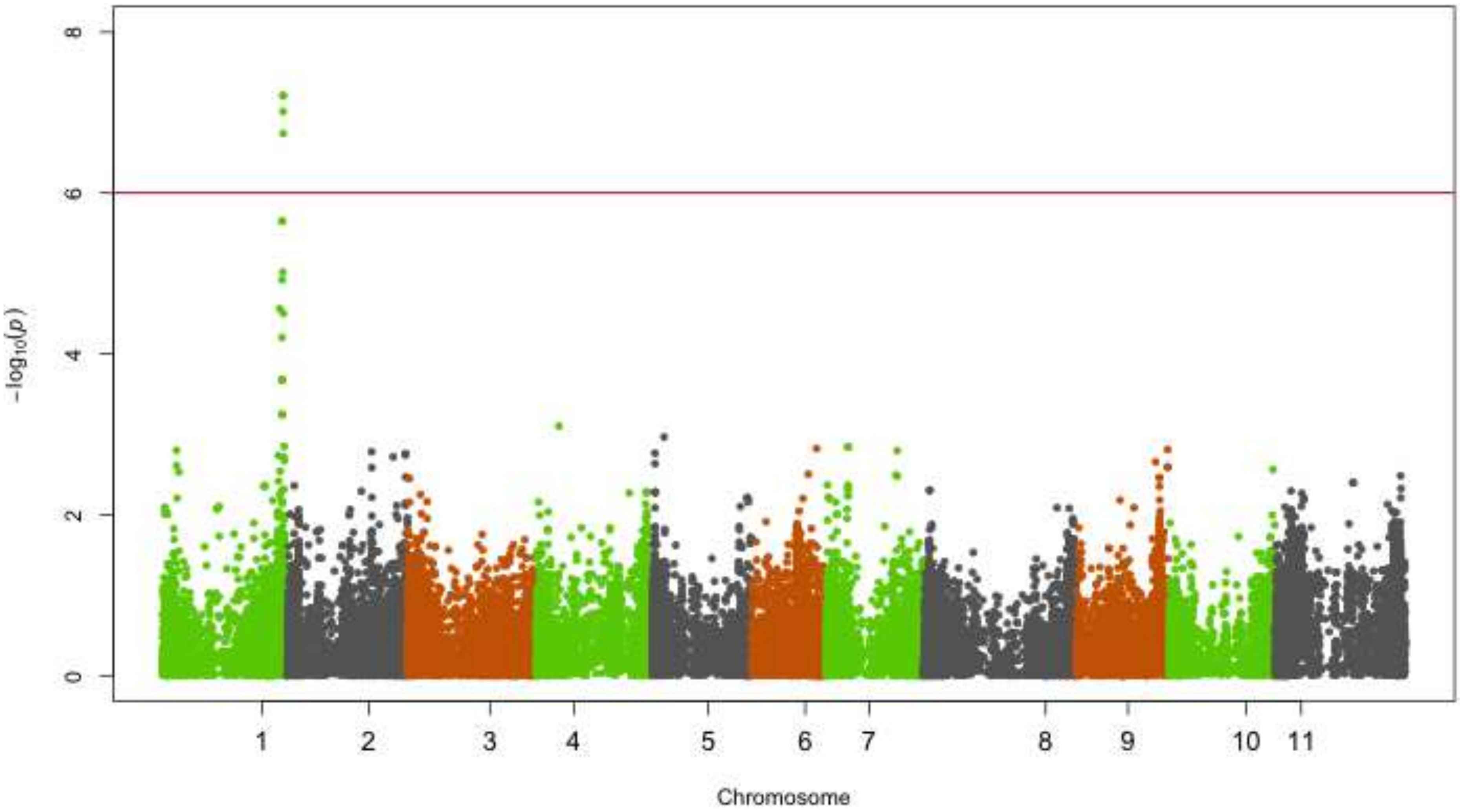

Figure

Click here to access/download;Figure;Fig 1.tiff

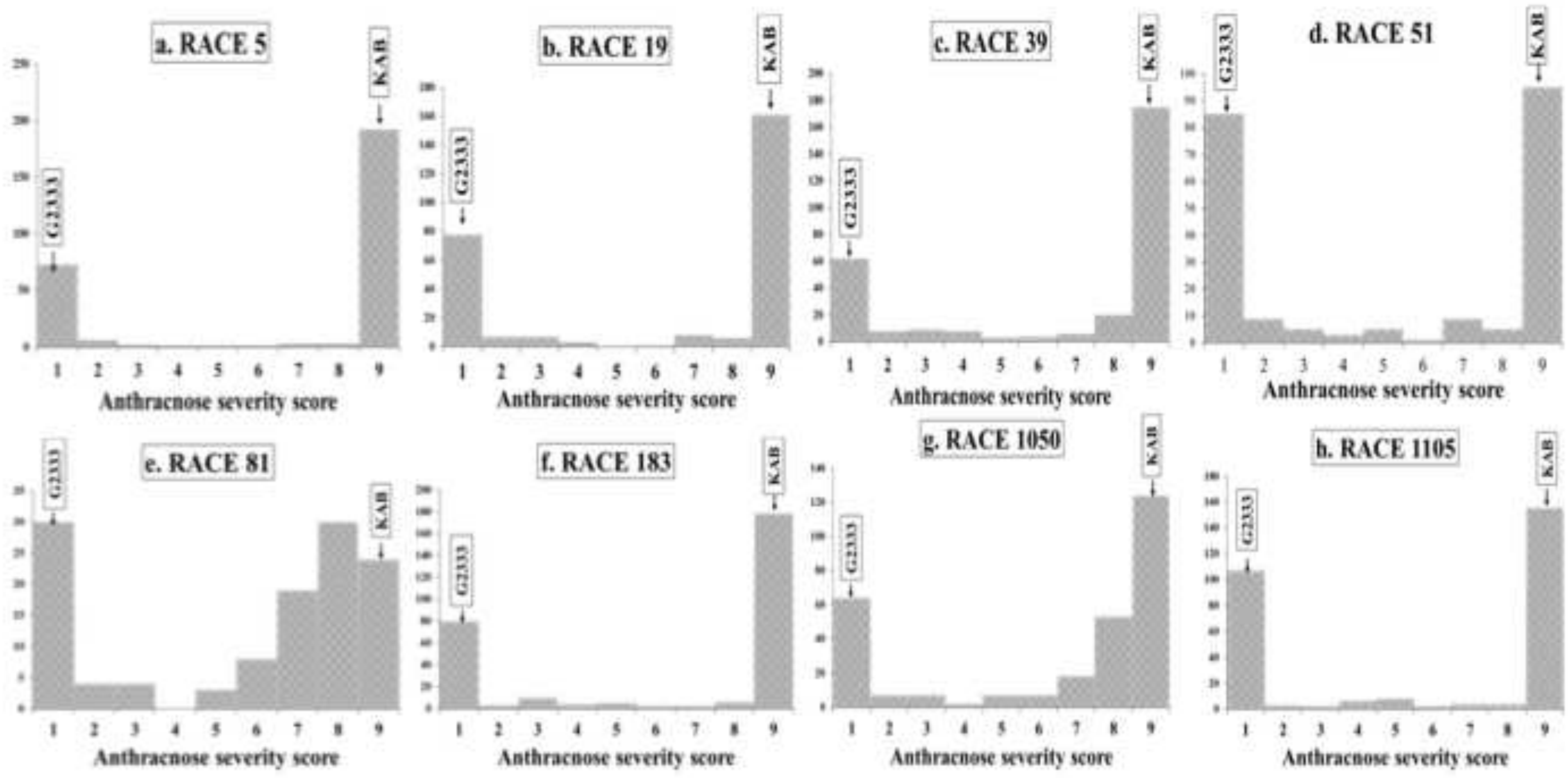

Click here to access/download
Supporting Information
S1 Table.xlsx

Revised Manuscript with Track Changes

Genome-wide association analysis of anthracnose resistance in the Yellow Bean Collection of Common Bean

Kuwabo Kuwabo[1], Swivia M. Hamabwe[1], Paul Kachapulula[1], Karen Cichy[2], Travis Parker[3], Chikoti Mukuma[4] and Kelvin Kamfwa[1*]

[1]Dept. of Plant Sciences, University of Zambia, Great East Road, Lusaka, Zambia.

[2]USDA-ARS, Sugarbeet and Bean Research Unit, 1066 Bogue St., East Lansing, MI 48824, USA.

[3]Dept. of Plant Sciences / MS1, Section of Crop & Ecosystem Sciences, University of California, 1 Shields Avenue, Davis, CA 95616-8780, USA.

[4]Zambia Agricultural Research Institute, Kasama, Zambia.

*Corresponding author

E-mail: kelvinkamfwa@gmail.com

Abstract

Anthracnose caused by *Colletotrichum lindemuthianum* is a major disease of common bean (*Phaseolus vulgaris*) worldwide. Yellow beans are a major market class of common bean especially in eastern and southern Africa. The objectives of this study were i) evaluate the yellow bean collection of 255 genotypes for resistance to races 5, 19, 39, 51, 81, 183, 1050 and 1105 of *C. lindemuthianum*.~~eight races of *C. lindemuthianum*~~, and ii) conduct genome-wide association analysis to identify genomic regions and candidate genes associated with resistance to~~ ten races of~~ *C. lindemuthianum*. ~~The Yellow Bean Collection comprised of 255 diverse yellow bean genotypes was evaluated for resistance to races 5, 19, 39, 51, 81, 183, 1050 and 1105 of *C. lindemuthianum*.~~ The Yellow Bean Collection was genotyped with 72,866 SNPs using Genotyping by Sequencing, and genome-wide association analysis was conducted using Mixed Linear Model in TASSEL. ~~Several~~ Andean and Middle American genotypes with superior levels of resistance to the eight races used in the current study were identified. The genotype YBC278 was the only one among 255 genotypes that was highly resistant to all eight races. Resistance in the Yellow Bean Collection to the eight races used in the current study was controlled by major-effect loci on chromosomes Pv01, Pv03, Pv04, Pv05 and Pv07. The genomic region on Pv01, which overlapped with the Andean locus *Co-1* provided resistance to races 81, 1050 and 1105. Significant SNPs for resistance to race 39 were identified on Pv02. The genomic region on Pv04, which ~~overlap~~overlapped~~s~~ with known major-effect loci *Co-3*, *Co-15*, *Co-16*, *Co-y* and *Co-z*, provided resistance to races 5, 19, 51 and 183. Novel genomic regions for resistance to race 39 were identified on Pv05 and Pv07. Plant

resistance genes (R genes) with NB-ARC and LRR domains, which occurred in clusters, were identified as positional candidate genes for genomic regions on Pv02 and Pv04.

Introduction

Common bean (*Phaseolus vulgaris*) is a source of food and nutritional security for millions of households in Latin America and Africa [1]. Beans play an important role in combating malnutrition because of its richness in protein, carbohydrates and minerals (especially iron and zinc) [2].

Beans have a rich diversity in traits such as seed color, seed size and seed shape [3]. These traits have been used to classify beans in different market classes. One of the market classes is the yellow bean, which is comprised of both Andean and Middle American genotypes. Within the yellow bean market class, there are several seed types classifications based on the shade of yellow color. These include the Manteca, Canary (Canario), Mayocoba, and Njano classes [4, 5]. Various yellow bean market classes are commercially important in Latin America, the Caribbean, and Africa [6, 7]. In Africa, yellow beans are popular in Angola, Kenya, Tanzania and Zambia [7]. In Zambia, the Manteca yellow landrace known as Lusaka is preferred over other bean market classes and fetches higher market prices [8]. In Tanzania, the Njano class is the most popular yellow seed type and it is estimated that about 32% of total bean production there is for yellow beans, and mainly the Njano type [9]. Yellow beans have become increasingly important bean corridors of both East and Southern Africa [10].

Consumers prefer y~~Y~~ellow beans to~~are preferential to~~ other market classes because of their superiority in several end-use traits. Manteca yellow beans~~ often~~ have shorter cooking time and higher iron bioavailability than other market classes [11-14]. Despite the consumer preference and nutritional attributes of some yellow bean market classes ~~hey~~ ~~–~~have historically received less breeding efforts compared to other popular bean market classes.

Average yields for yellow bean are often lower than other bean classes. Several abiotic and biotic stresses contribute to low yields [15]. In some countries, continued use of yellow bean landraces with poor genetics and yield potential contribute to low yields. Diseases are a major contributing factor to the low yields of yellow beans. Anthracnose (ANTH) (caused by *Colletotrichum lindemuthianum*) is a major disease that affects yellow beans. Depending on varietal susceptibility and weather conditions (high humidity and moderate temperatures favors its spread), ANTH can cause yield losses of up to 100% [16]. ANTH is a seed borne disease and planting infected seed is a major mode of its transmission [17, 18]. This transmission mode of ANTH makes its control/or management challenging because of the widespread use of on-farm saved seed in Africa and Latin America. Though ANTH can effectively be controlled using fungicides, smallholder farmers who are the majority of bean growers cannot afford fungicides. Additionally, there are health and safety concerns involved in using fungicides. Development and use of resistant varieties is the most cost-effective and ecologically-sound management strategy for anthracnose~~ANTH~~.

ANTH resistance exists within the primary gene pool of common bean and several sources of resistance have been identified and used in breeding. Major-effect genes mainly control resistance to ANTH in common bean, though~~ a few~~ minor QTL have also been identified [19,20]. To date, 19 dominant major-effect ANTH resistance genes have been identified in common bean~~on seven chromosomes~~. These genes are classified as either Andean or Middle American depending on whether they were identified in an Andean or Middle American genotype. Andean genes include *Co-1, Co-12, Co-13, Co-14, Co-15, Co-x, Co-w, Co-y*, and *Co-z* while Middle American genes include *Co-2, Co-3, Co-4, Co-5, Co-6, Co-11*, *Co-16*, *Co-17*, *Co-u*, and *Co-v* [21~~19~~-24~~2~~]. The genes *Co-1*, *Co-3, Co-4* and *Co-5* are multi-allelic. Some of these major genes are located in genomic regions that contain clusters of disease resistance genes that control resistance to multiple diseases [25~~3~~, 26~~4~~]. For example, *Co-1* is located in the genomic that also contain *Pgh-1*, which is a major gene for resistance to angular leaf spot. In addition to the major genes, QTL with smaller effect on ANTH resistance have been reported [27~~5~~].

The major challenge in breeding for durable ANTH resistance is the broad genetic diversity of *Colletotrichum lindemuthianum*. To date, over 147 races of *C. lindemuthianum* have been reported worldwide [28~~6~~]. *C. lindemuthianum* races have co-evolved with the two gene pools of common beans and are classified as either Andean of Middle American [21~~19~~]. Andean races are virulent mostly on Andean beans while Middle American races are virulent on both Andean and Middle American beans. Because of the diversity of *C. lindemuthianum*, resistance often breaks down; therefore, there is need for continuous identification of new sources of resistance and understanding

the genetic architecture of that resistance. M~~Resistance to ANTH of the m~~ajor genes for ANTH resistance follow~~s~~ ~~the~~ gene-for-gene disease resistance concept. Therefore, some genes are effective against some races, but less effective against others. It is therefore important to identify genes that are effective against specific races so as to develop varieties with effective and durable resistance for a given geographic location. Diversity panels are important genetic resources that can serve as sources of resistance and also for understanding the genetic architecture of traits such as *C. lindemuthianum*. The Yellow Bean Collection (YBC) is one of the diversity panels that have been assembled in common bean [4]. The YBC~~is panel~~ is comprised of landraces, varieties and elite lines of yellow beans, which are variable for several traits including anthracnose resistance. The YBC, therefore, is an excellent genetic resource for understanding the extent of ANTH resistance and its genetic architecture in the yellow bean market class.

QTL mapping using recombinant inbred lines and genome-wide association studies (GWAS) using diversity panels are two commonly used genetic approaches to understand the genetic architecture of traits. Both approaches have previously been used to identify genomic regions for resistance to variable races of *C. lindemuthianum* in common bean. One of the advantages of GWAS over QTL mapping is the higher mapping resolution that it offers, as GWAS takes advantage of historical recombination, leading to smaller linkage disequilibrium blocks. The enhanced mapping resolution of GWAS offers a better opportunity to precisely identify candidate genes for resistance to anthracnose. Additionally, GWAS using diversity panels provide an opportunity to identify minor effect QTL~~ because they are relatively larger in size~~ that QTL mapping populations using

recombinant inbred lines cannot identify. Minor ~~effect~~ QTL are important sources of horizontal resistance, and if put in the same genetic background with major effect genes, which offers vertical resistance, they can confer durable resistance to multiple races of *C. lindemuthianum*. The objectives of this study were i) evaluate the yellow bean collection for resistance to eight races of *C. lindemuthianum*, and ii) conduct genome-wide association analysis to identify genomic regions and candidate genes associated with resistance to eight races of *C. lindemuthianum*.

Materials and methods

Plant materials

In the current study the Yellow Bean Collection (YBC) ~~comprised~~ of 255 genotypes consisting of landraces, breeding lines and varieties with variable shades of yellow seed colors from Africa, North America, South America, Europe, Central America, Caribbean, Middle East and East Asia was used [4]~~genotypes was used. Details on the composition of the YBC can be found in [4]. Briefly, the YBC is comprised of landraces, breeding lines and varieties with variable shades of yellow seed colors from various countries and breeding programs~~. These shades of yellow seed colors include Amarillo dark and light, beige, brown, Canary (Mexican), Manteca, Mayocoba, and green-yellow (Njano). The YBC has both Andean and Middle American genotypes.

~~The YBC was evaluated for resistance to eight characterized races of *C. lindemuthianum*.~~

Evaluation of the YBC for resistance to anthracnose

A total of eight races (5, 19, 39, 81, 183, 1050 and 1105) of *C. lindemuthianum* were used in the current study. These eight races were characterized from isolates collected from major bean-growing regions of Zambia ~~. Characterization was conducted~~ using a set of 12 differential cultivars [29~~7~~]. The eight races used in the current study offered a broad spectrum of virulence. Inoculation of the YBC followed a protocol described by~~in~~ Mungalu et al. 2020~~[28]~~. This protocol involved planting the YBC (255 genotypes), anthracnose susceptible check (Kabulangeti) and anthracnose resistant check (G2333) on Styrofoam trays~~. Each tray had 60 wells and each well was 5 cm wide, 5 cm long and 5 cm deep~~. A completely randomized design with three replications was used. Each replication had two seedlings in a well. Therefore, a total of six seedlings per genotype were evaluated for resistance to each race. Inoculation was conducted when seedlings had developed fully expanded unifoliate leaves. Evaluation of the YBC was conducted separately for each of the eight races.

Inoculum preparation

Inoculum preparation was conducted using a protocol described in Mungalu et al. 2020~~[28]~~. Each race was cultured on petri dishes on either potato dextrose agar (PDA) 39 g L^{-1} or modified Mathur's agar culture media made with dextrose (8 g L^{-1}), $MgSO_4 \cdot 7H_2O$ (2.5 g L^{-1}), KH_2PO_4 (2.7 g L^{-1}), neopeptone (2.4 g L^{-1}), yeast extract (2.0 g L^{-1}), and agar (16 g L^{-1}). ~~The reason for using either PDA or Mathur's agar was because some isolates sporulated better on PDA while others on Mathur's agar.~~ The petri dishes were incubated in the dark at 23-25 ºC for 7 to 10 days for sporulation. Sporulated plates where flooded with distilled water and allowed to settle~~it~~ for 20 minutes. Spores where

Formatted: Subscript
Formatted: Subscript
Formatted: Subscript
Formatted: Subscript

then harvested by scrapping them off from the culture using a glass rod. The harvested spore suspension was then sieved through a double-layered cheesecloth into a clean beaker. The spore suspension concentration was then adjusted to 1.2 x 10^6 spores per ml using a hemocytometer as described in Mungalu et al. 2020. Tween 20 was added to the inoculum for adhesion. Seedlings were thoroughly sprayed with the inoculum on both surfaces of the primary leaves and the stem using a hand sprayer. After inoculation, plants were left to air-dry for a few minutes before they were placed in a high humidity chamber (>90% humidity) for 72 hrs at room temperature. After this incubation seedlings were removed from the humidity chamber and transferred to greenhouse benches where they stayed for 5 days for further anthracnose development. Anthracnose severity on seedlings was rated based on a 1-9 CIAT scale [3429]. The score range of 1-3 was considered as resistant and included plants with no visible symptoms or with few very small lesions, mostly on primary leaf veins, 4-6 was considered moderately resistant and included seedlings with small lesions on leaves and seedling stem, and 7-9 was considered susceptible and included dead seedlings and those with numerous small or enlarged lesions, with sunken cankers on leaves and seedling stem.

Severity score analyses

Statistical analyses on disease severity scores were conducted in SAS 9.3 [350]. Severity score data was first checked for normal distribution using PROC UNIVARIATE. Normality test results showed that severity scores for all races were normally distributed. Analysis of Variance was conducted using PROC MIXED following the mixed model:

$Y_{ik}=\mu+\alpha_i+\gamma_k+E_{ik}$

Where: Y_{ik} was anthracnose severity score, with genotype i, replication k; αi was the fixed variable effect of the genotype i; γ was the random variable effect of a replication; ε was the residual associated with replication k in genotype i. γk was a random variable, which was assumed to be normally distributed with mean = 0.

Genotypic data analyses

The YBC ~~(255 genotypes)~~ was genotyped with 72,866 Single Nucleotide Polymorphism (SNPs) markers using Genotyping by Sequencing (GBS). The -DNA sequencing libraries were prepared using a single restriction enzyme ApeKI. The Genomics Core at Michigan State University sequenced the libraries using an Illumina standard HiSeq 4000 to generate 150 bp single-end reads.~~at Michigan State University in the USA.~~ Additional d~~D~~etails on ~~this~~ genotyping can be found in Sadohara et al. 2022 ~~[4]~~. The SNP data was used in population structure, kinship and SNP-disease severity analyses.

Population structure

Correction for population structure in a diversity panel such as the YBC is necessary to avoid false positives in GWAS. ~~Details on the population structure in the YBC can be found in [4].~~ In the current study, the population structure in the YBC was determined using Principal Component Analysis (PCA) in TASSEL v.5.0 [36~~1~~]. The first five principal components, which together accounted for 62.5% of variation in the YBC, were used as covariates in the GWAS analyses.

Kinship

Kinship (cryptic relatedness) among genotypes in a diversity panel such as the YBC can result on false positive. In the current study, kinship among genotypes was investigated and corrected for in GWAS using the Identical by Descent method in TASSEL.

Association analysis for anthracnose resistance

SNPs significantly associated with resistance to races 5, 19, 39, 51, 81, 183, 1050 and 1105 were identified using the following Mixed Linear Model [372]:

$$Y = G + P + K + \varepsilon$$

Where Y the phenotype of a genotype; X was the fixed effect of the SNP; P was the fixed effect of population structure (from PCA matrix from TASSEL); K was the random effect of relative kinship (from kinship matrix from TASSEL); ε was the error term, which was assumed to be normally distributed with mean = 0. MLM analysis was conducted in the software TASSEL v.5.0. Before use in MLM, SNP data was filtered for minor allele frequency (MAF=5%), which reduced the number of usable SNPs for MLM to 55,000. The Bonferroni-corrected *P*-value of 1.0×10^{-6} ($\alpha = 0.05$; 50,000 SNPs) was used as a threshold to determine the significance of the association between the SNP and severity score of a given race. The Manhattan plots for visualization of significant SNPs were made using a custom R script.

Identification of candidate genes

Candidate genes in genomic regions associated with resistance to the eight races of ANTH in the current study were identified from *Phaseolus vulgaris* v2.1 [383] in Phytozome using JBrowse following a previously described method [394]. Briefly, a gene was identified as a positional candidate gene based on two criteria: (i) it was within a genomic region of 400 kb of either upstream or downstream of the most significant SNP, and (ii) the functional role of a gene in disease resistance has been determined or proposed.

Results

Anthracnose severity score analysis

Highly significant (*P*<0.01) differences were observed among YBC genotypes in their reaction to races 5, 19, 39, 51, 81, 183, 1050 and 1105 of *C. lindemuthianum*. Severity scores for the YBC ranged from 1 to 9 for all eight races (Table 1).

Table 1. Means of anthracnose severity score of G2333 (resistant check), Kabulangeti (susceptible check) and 255 lines of the YBC evaluated for resistance in the greenhouse at University of Zambia, Lusaka, Zambia.

	Checks		YBC		
**ANTH race**	**G2333**	**Kabulangeti**	**Mean**	**Range**	**ANOVA**
**Race 5**	1.0	9.0	6.7±0.2	1.0-9.0	***
**Race 19**	1.0	9.0	6.3±0.21	1.0-9.0	***
**Race 39**	1.0	9.0	6.7±0.19	1.0-9.0	***
**Race 51**	1.0	9.0	5.2±0.21	1.0-9.0	***
**Race 81**	1.0	9.0	5.9±0.18	1.0-9.0	***

**Race 183**	1.0	9.0	6.4±0.2	1.0-9.0	***
**Race 1050**	1.0	9.0	6.5±0.19	1.0-9.0	***
**Race 1105**	1.0	9.0	5.7±0.21	1.0-9.0	***

*** = Signifiacant ($p < 0.001$)

The average severity score for the YBC to races 5, 19, 39, 51, 81, 183, 1050 and 1105 were 6.7, 6.3, 6.7, 5.2, 5.9, 6.4, 6.5 and 5.7, respectively (Table 1). The highest mean severity scores for the YBC were observed for Andean races 5 and 39. The frequency distribution of severity scores showed a bimodal distribution for all eight races (Fig 1).

Fig 1. Frequency distributions of severity scores for races 5, 19, 39, 51, 81, 183, 1050 and 1105 of *C. lindemuthianum* inoculated on the Yellow Bean Collection, resistant check (G2333) and susceptible check (Kabulangeti)~~eight races 5, 19, 39, 51, 81, 183, 1050 and 1105 of *C. lindemuthianum*~~.

The resistant and susceptible checks reacted to all eight races as expected. The resistant (G2333) and susceptible (Kabulangeti) checks consistently scored 1 and 9, respectively, for all eight races (Table 1). Of the 255 YBC genotypes evaluated, five genotypes (YBC278, YBC130, YBC173, YBC192 and YBC267) showed superior resistance to most of the eight races (Table 2).

Table 2. Mean anthracnose severity scores for the five genotypes identified from the 255 Yellow Bean Collection genotypes as being highly resistant against races 5, 19, 39, 51, 81, 183, 1050 and 1105 of *Colletotrichum lindemuthianum* after artificial inoculation in the greenhouse at University of Zambia, Lusaka, Zambia.

YBC ID	ID	Country	Seed color	Genepool	Mean anthracnose scores							
					Race 5	Race 19	Race 39	Race 51	Race 81	Race 183	Race 1050	Race 1105
**YBC278**	SMC28	Uganda	Amarillo	Middle-American	1.0	1.0	1.0	1.0	1.0	1.0	1.0	1.0
**YBC130**	P1527538	Burundi	Green yellow	Andean	1.0	1.0	-	1.2	1.0	1.0	1.0	1.0
**YBC173**	G22501	Burundi	Green yellow	Andean	1.0	1.0	9.0	1.0	1.0	1.0	1.3	1.0
**YBC267**	DAB933	Uganda	Amarillo	Andean	3.3	1.0	1.0	1.0	1.3	3.5	-	1.0
**YBC192**	Roba-1	Uganda	Amarillo	Middle-American	1.0	1.3	-	1.0	6.8	1.0	1.7	1.0

The genotype YBC278 was the only one that that was highly resistant (severity score < 1) to all eight races. The severity scores for all 255 YBC genotypes for the eight races are presented in S1 Table.

Genome-wide association analysis

SNPs significantly associated with resistance to races 5, 19, 39, 51, 81, 183, 1050 and 1105 were identified on chromosomes Pv01, Pv02, Pv04, Pv05 and Pv07 (Fig 2,-9 , Table 3).

Fig 2. Manhattan plot showing SNPs on chromosome Pv01 significantly associated with resistance to race 81 of the Yellow Bean Collection genotypes evaluated in the greenhouse at University of Zambia, Lusaka, Zambia. The red solid horizontal line is the Bonferroni adjusted *P*-value (1.0E-06).

Fig 3. Manhattan plot showing SNPs on chromosome Pv01 significantly associated with resistance to race 1050 of the Yellow Bean Collection genotypes evaluated in the greenhouse at University of Zambia, Lusaka, Zambia. The red solid horizontal line is the Bonferroni adjusted *P*-value (1.0E-06).

Fig 4. Manhattan plot showing SNPs on chromosome Pv01 significantly associated with resistance to race 1105 of the Yellow Bean Collection genotypes evaluated in the greenhouse at University of Zambia, Lusaka, Zambia. The red solid horizontal line is the Bonferroni adjusted *P*-value (1.0E-06).

Fig 5. Manhattan plot showing SNPs on chromosome Pv02, Pv04, Pv05 and Pv07 significantly associated with resistance to Andean ANTH race 39 of the Yellow Bean Collection genotypes evaluated in the greenhouse at University of Zambia, Lusaka, Zambia. The red solid horizontal line is the Bonferroni adjusted *P*-value (1.0E-06).

Fig 6. Manhattan plot showing SNPs on chromosome Pv04 significantly associated with resistance to race 19, 51 and 183 of the Yellow Bean Collection genotypes evaluated in the greenhouse at University of Zambia, Lusaka, Zambia. The red solid horizontal line is the Bonferroni adjusted *P*-value (1.0E-06).

Fig 7. Manhattan plot showing SNPs on chromosome Pv04 significantly associated with resistance to race 51 of the Yellow Bean Collection genotypes evaluated in the greenhouse at University of Zambia, Lusaka, Zambia. The red solid horizontal line is the Bonferroni adjusted *P*-value (1.0E-06).

Fig 8. Manhattan plot showing SNPs on chromosome Pv04 significantly associated with resistance to race 183 of the Yellow Bean Collection genotypes evaluated in the greenhouse at University of Zambia, Lusaka, Zambia. The red solid horizontal line is the Bonferroni adjusted *P*-value (1.0E-06).

Fig 9. Manhattan plot showing SNPs on chromosome Pv04 significantly associated with resistance to Andean ANTH race 5 in the Yellow Bean Collection evaluated in

the greenhouse at University of Zambia, Lusaka, Zambia. The red solid horizontal line is the Bonferroni adjusted *P*-value (1.0E-06).

Table 3. **SNPs markers significantly associated with resistance to races 5, 19, 39, 51, 81, 183, 1050 and 1105 of *Colletotrichum lindemuthianum* identified using the Yellow Bean Collection evaluated in the greenhouse at University of Zambia, Lusaka, Zambia**.

**Trait**	**Chr**	**SNP**	**Position (bp)**	***P*-value**	**R^2 (%)**
**Race 5**	Pv04	Chr04_40179029	4,0179,029	1.5E-06	12.6
**Race 19**	Pv04	Chr04_2140558	2,140,558	1.1E-11	24.7
**Race 39**	Pv02	Chr02_49318523	49,318,523	7.4E-10	30.1
**Race 39**	Pv05	Chr05_15211384	15,211,384	1.19E-07	13.3
**Race 39**	Pv07	Chr07_27213163	27,213,163	7.15E-07	14.2
**Race 51**	Pv04	Chr04_2140558	2,140,558	7.6E-10	18.8
**Race 81**	Pv01	Chr01_49584097	49,584,097	6.2E-08	41.2
**Race 183**	Pv04	Chr04_1067693	1,067,693	3.4E-11	22.5
**Race 1050**	Pv01	Chr01_49583965	49,583,965	1.9E-12	27.5
**Race 1105**	Pv01	Chr01_49584097	49,584,097	1.1E-10	21.4

Chr = Chromosome; bp = base pairs; *P*-value = level of significance; E = exponential; R^2 = proportion of variation of anthracnose resistance explained by the significant SNP

Chromosome Pv01

Several SNPs in the genomic region spanning 49,151,104 bp - 49,584,097 bp on chromosome Pv01 were significantly associated with resistance to races 81, 1050 and 1105 (Fig 2 - 4). The most significant SNP (Chr01_49584097) in this genomic region

explained 41.2%, 27.5% and 21.4% of the YBC variation in ANTH severity caused by races 81, 1050 and 1105, respectively (Table 3).

Chromosome Pv02

Significant SNPs for resistance to the Andean ANTH race 39 were identified on Pv02 in a genomic region spanning from 49,155,240 bp - 49,416,064 bp (Fig 5). The most significant SNP (Chr02_49318523) in this genomic region explained 30.1% of the YBC variation in ANTH severity caused by race 39 (Table 3).

Chromosome Pv04

Significant SNPs for races 19, 51 and 183 were identified in separate genomic regions of Pv04 (Fig 6 - 8). The first genomic region was at the beginning (1,067,693 bp - 2,140,558 bp) of Pv04. Several SNPs in this genomic region were associated with resistance to races 19, 51 and 183. The R^2 for the most significant SNP (–Chr04_2140558, Chr04_2140558 and Chr04_1067693) for races 19, 51 and 183 were 24.7%, 18.8% and 22.5%, respectively. The second genomic region identified on Pv04 was at 40,179,029 bp. This region was significantly associated with resistance to race 5 and the most significant SNP at this region explained 12.6% of the variation in the YBC for ANTH severity caused by race 5.

Chromosome Pv05 and Pv07

SNPs significantly associated with resistance to race 39 were identified on Pv05 and Pv07 (Figu 9). The most significant SNPs on Pv05 (Chr05_15211384; 15,211,384 bp)

and Pv07 (Chr07_27213163; 27,213,163 bp) explained 13.3% and 14.2% of the variation in severity scores for race 39 (Table 3).

Discussion

Anthracnose caused by *C. lindemuthianum* is a major disease of common bean. The productivity of yellow beans, a major market class in several African countries, is constrained by anthracnose. In the current study, we evaluated the YBC for its reaction to races 5, 19, 39, 51, 81, 183, 1050 and 1105 of *C. lindemuthianum*, and conducted GWAS to identify genomic regions and positional candidate genes conferring resistance to these eight races.

The frequency distribution graphs for severity for all eight races showed a bimodal pattern, suggesting that major-effect genes were the basis of ANTH resistance, which is consistent with previous description of ANTH resistance in common bean as being controlled by major-effect genes. Identification of sources of resistance to anthracnose within the yellow bean market class would necessitate progress in the genetic enhancement of yellow beans for resistance to anthracnose. Such identification is necessary because of the challenges associated with poor recovery of yellow seed color and seed size in progenies from crosses of yellow bean with other colors. The current study has confirmed the existence of adequate levels of ANTH resistance to both Andean and Middle-American races of *C. lindemuthianum* in the yellow bean market class. In this study the genotype YBC278 was identified as having superior resistance to ANTH. YBC278 was the only genotype in the YBC that was highly resistant (severity score of

less than 1) to all the eight races used in the current study. This reaction was only similar to that of the resistant check G2333, a Middle-American genotype widely reported to be highly resistant to both Andean and Middle-American races of *C. lindemuthianum*. YBC278, also known as SMC28, is a Middle American bean of race Mesoamerica from CIAT-Uganda. It has an Amarillo (dark yellow) seed type and a 100-seed weight of 27 grams. The identification of YBC278 as highly resistant to both Middle American and Andean genotypes is important, as it will serve as a source of superior resistance for developing yellow bean varieties with durable resistance to anthracnose. Genotypes YBC130 and YBC267 are the other two genotypes that showed superior levels of resistance to ANTH. YBC130 is an Andean landrace from Burundi with a green-yellow seedcoat and a 100-seed weight of approximately 43 grams. It was included in the Andean Diversity Panel as ADP0468 and is listed as PI 527538 in the USDA-NPGS gene bank. YBC267, also known as DAB 933, is an Andean bean from CIAT-Uganda of the Amarillo (dark) market category, with a 100-seed weight of 30 grams. These accessions could be used as alternative sources of ANTH resistance for yellow bean breeding.

The genetic basis of the observed resistance in the YBC to eight races of *C. lindemuthianum* was explored using genome-wide association analysis. Genomic regions associated with resistance to the eight races used in the current study were identified on chromosomes Pv01, Pv02, Pv04, Pv05 and Pv07.

Several SNPs within a 528 kbp region (49,151,104-49,679,631 bp) on Pv01 were significantly associated with resistance to race 81, and the highly virulent races 1050 and

1105. This is a major-effect QTL and it appears to be the major locus *Co-1*, which confers resistance mainly to Middle American races of *C. lindemuthianum*. The SNPs most significantly associated with resistance to races 81, 1050, and 1105 were found at positions 49,584,097, 49,583,965, and 49,584,097 of Pv01, respectively. In each case, the most SNP most significantly associated with ANTH resistance is in the gene model Phvul.001G243800, a protein-kinase recently proposed to underlie *Co-1* [40,4135]. This is a powerful validation of the role of this gene, as well as the experimental methods employed in this study. Previous studies have shown existence of both Andean and Middle American races in East and Southern African countries including Zambia, which underscores the importance of having *Co-1* gene in Andean genotypes such as the yellow beans.

The genomic region 49,318,523 bp on Pv02 contained SNPs that were significantly associated with resistance to races 19 and 39. The identification of YBC genotypes resistant to races 19 and 39, and their associated resistance genomic regions has breeding significance to Zambia and other bean producing countries for two reasons. First, race 19 is the most prevalent race in the bean-producing regions of Zambia. Second, race 39 is a highly virulent Andean race [275] and it is important that yellow bean genotypes with superior resistance to race 39 are identified to serve as source of resistance for genetic improvement of yellow beans in countries where it is a major market class. The association between race 39 and the genomic region on Pv02 (49,318,523 bp) has previously been reported [275]. Eight plant disease resistance genes (R genes) including Phvul.002G323100, Phvul.002G323200, Phvul.002G323300, Phvul.002G323400,

Phvul.002G323404, Phvul.002G323704, Phvul.002G323708, and Phvul.002G323712 with NB-ARC and LRR domains were identified as positional candidate genes for resistance to race 39 identified on Pv02. R genes encode receptors that play a key role in the recognition of avirulence (Avr) products expressed by the pathogen during infection, which triggers an immune response and is the basis for gene-for-gene or race-specific resistance for ANTH observed in common bean.

The genomic region on 1.1 Mbp – 2.1 Mbp on Pv04 conferred resistance to races 19, 51 and 183. This genomic region overlaps with major-effect loci *Co-3*, *Co-15*, *Co-16*, *Co-y* and *Co-z*. The relatively large number of races whose resistance was controlled by this genomic is indicative of its significant role in the YBC as a source of broad-spectrum resistance to multiple races of *C. lindemuthianum*. The genomic region 1.1 Mbp – 2.1 Mbp identified in the current study as providing resistance to races 19, 51 and 183 was previously reported to also provide resistance to races 7, 19, 49, 55, 109, 530, 566 and 1331 [275, 3028], which underscores the importance of this genomic region for resistance to multiple races of anthracnose. Five disease resistance (R) genes including Phvul.004G015600, Phvul.004G015800, Phvul.004G015900, Phvul.004G016000 and Phvul.004G016532 with the NB-ARC and LRR domains were identified as candidate genes for resistance to races 19, 51 and 183 at the 1.1 Mbp - 2.1 Mbp genomic region on Pv04. These R genes occurred in a cluster and it is plausible that some of them provide race-specific resistance because this genomic region provided resistance to a relatively large number of races. Given that *Co-3* has previously been reported as multi-allelic it is plausible that resistance to races 19, 51 and 183 reported in the current study may be

conditioned by different alleles. In addition to overlapping with *Co-3*, the genomic region on Pv04 identified in the current study also overlaps with the telomere cluster of resistance genes for rust (*Ur-5* and *Ur-14*) [42~~36~~], angular leaf spot (*Phg-3*) [42~~36~~], powdery mildew [43~~37~~] and halo blight QTL (HB4.2) [44~~38~~], which are major diseases of beans in Africa. Given the convergence of resistance to several races of ANTH and other major diseases at the identified genomic region in the current study, it is a worthwhile target for Marker-assisted selection to develop common bean varieties with durable resistance to ANTH and also multiple resistances to ANTH, rust and angular leaf spot. The current study has identified another genomic region on Pv04 to control resistance to ANTH. This genomic region located at the distal end on Pv04 (40.2 Mbp) is a major locus (R^2=12.6%) and the basis for resistance in the YBC to race 5 (Figure 5). This is the first report of ANTH resistance to race 5 at this genomic region.

Significant SNPs for resistance to race 39 were identified on Pv05 (15.2 Mbp) and Pv07 (27.2 Mbp). Given the large R^2 for the most significant SNPs on Pv05 (13.1%) and PV07 (15.5%) the two QTL had major effect on resistance to race 39 in the YBC. This is the first report of resistance for race 39 on Pv05 and Pv07. Banoo et al. (2020) identified significant SNPs for resistance to races 87 and 73 on Pv05 and Pv07, but in a genomic region different from the one identified for race 39 in the current study. Resistance to race 39 has previously been reported on other chromosomes but not in the genomic regions on Pv05 and Pv05 identified in the current study. The locus *Co-2* on Pv11 was previously identified using a population of RILs as the source of resistance to race 39 [26~~4~~]. A previous GWAS conducted using the ADP reported resistance to race 39 only

on chromosome Pv04 [27~~5~~], the same region we have also identified in the current study. Our study has not only identified a previously known genomic region on Pv04 as source of resistance to race 39, but has also identified novel major QTL on Pv05 and Pv07 as additional genomic regions for resistance to race 39.

Conclusion

Three YBC accessions with superior resistance to races 5, 19, 51, 81, 183, 1050 and 1105 were identified. The accession YBC278 was notable because it is the only one in the YBC that showed strong resistance to all eight races of *C. lindemuthianum* used in the current study.

The genetic architecture for resistance to eight races of *C. lindemuthianum* in the YBC has been determined. Major-effect loci on Pv01 (*Co-1*), Pv02 and Pv04 (which overlaps with *Co-3*, *Co-13*, *Co-15*, *Co-y* and *Co-z*) controlled most of the observed resistance to eight races observed in the YBC. In addition to the identified major-effect loci genomic regions on Pv01, Pv02 and Pv04, novel genomic regions on Pv04 (40.2 Mbp), Pv05 (15.2 Mbp) and Pv07 (27.2 Mbp) were significantly associated with the Andean races 5 and 39. Clusters of R genes with NB-ARC and LRR domains on Pv02 and Pv04 were identified as candidate genes.

Acknowledgement

We thank the University of Zambia Bean Breeding Program team for their assistance in conducting inoculations. We also thank Sadohara of Dr. Karen Cichy's for DNA collection.

~~The Kirkhouse Trust of the United Kingdom through the African Bean Consortium Project provided scholarship to Mr. Kuwabo Kuwabo. The opinions expressed in this publication are those of the authors and do not necessarily reflect the views of the Kirkhouse Trust~~.

References

1. Uebersax MA, Cichy KA, Gomez FE, Porch TG, Heitholt J, Osorno JM, et al. Dry beans (*Phaseolus vulgaris* L.) as a vital component of sustainable agriculture and food security. A review. Legume Science. 2022; e155.
2. Haas J, Luna S, Lung'aho M, Wenger M, Murray-Kolb L, Beebe S, et al. Consuming iron bio-fortified beans increases iron status in Rwandan women after 128 days in a randomized controlled feeding trial. J Nutr 2016; 146: 1586-1592
3. Singh SP. Broadening the genetic base of common bean cultivars: a review. Crop Sci. 2001; 41(6): 1659-1675.
4. Sadohara R, Izquierdo P, Couto Alves F, Porch T, Beaver J, Urrea CA, et al. The *Phaseolus vulgaris* L. Yellow Bean Collection: genetic diversity and characterization for cooking time. Genet Resour Crop Evol. 2022; 69(4): 1627-1648

5. Sadohara R, Long Y, Izquierdo P, Urrea CA, Morris D, Cichy K. Seed coat color genetics and genotype x environment effects in yellow beans via machine-learning and genome-wide association. Plant Genome. 2021; 15(1): e20173
6. Voysest O. Yellow beans in Latin America. Ann Rep Bean Improv Coop. 2021; xii–xviii.
7. Buruchara R, Chirwa R, Sperling L, Mukankusi C, Rubyogo JC, Mutonhi R, et al. Development and delivery of bean varieties in Africa: the Pan-Africa Bean Research Alliance (PABRA) model. Afr Crop Sci J. 2011; 19(4): 227-245.
8. Sichilima T, Mapemba L, Tembo, G. Drivers of dry common beans trade in Lusaka, Zambia: A trader's perspective. Sustain Agric Res. 2016; 5(2): 15-26.
9. Sperling L, Birachi E, Kalemera S, Mutua M, Templer N, Mukankusi C, et al. The informal seed business: focus on yellow bean in Tanzania. Sustainability. 2021; 13(16): 8897
10. Farrow A, Muthoni-Andriatsitohaina R, editors. Atlas of common bean production in Africa 2nd ed. Nairobi, Kenya: 2022.
11. Wiesinger JA, Cichy KA, Tako E, Glahn RP. The fast cooking and enhanced iron bioavailability properties of the Manteca yellow bean (*Phaseolus vulgaris* L.). Nutrients. 2018; 10(11): 1609.
12. Hart JJ, Tako E, Wiesinger J, Glahn RP. Polyphenolic profiles of yellow bean seed coats and their relationship with iron bioavailability. Agric Food Chem. 2019; 68(3): 769-778.

13. Cichy KA, Wiesinger JA, Mendoza FA. Genetic diversity and genome-wide association analysis of cooking time in dry bean (*Phaseolus vulgaris* L.). Theor Appl Genet. 2015; 128(8): 1555-1567.

14. Mishili FJ, Temu A, Fulton J, Lowenberg-DeBoer J. Consumer preferences as drivers of the common bean trade in Tanzania: A marketing perspective. J Int Food Agribus Mark. 2011; 23(2): 110-127.

15. Parker TA, Acosta Gallegos J, Beaver J, Brick M, Brown JK, et al. Genetic Resources and Breeding Priorities in Phaseolus Beans: Vulnerability, Resilience, and Future Challenges. In: Plant Breeding Reviews. 2023; 46: 289.

16. Pastor-Corrales MA, Tu JC. Anthracnose. In: H.F. Schwartz, and M.A. Pastor-Corrales, editors. Bean production problems in the tropics. CIAT, Colombia; 1989. p 77–104

17. Schwartz HF, Pastor-Corrales MA. Anthracnose. In: Schwartz HF, Steadman JR, Hall R, and Forster RL, editors. Compendium of Bean Diseases. St. Paul Minnesota: APS Press; 2005. p. 25–27.

18. Ferreira JJ, Campa A, Kelly JD. Organization of genes conferring resistance to anthracnose in common bean. In: Varshney RK, Tuberosa R, editors. Translational Genomic for Crop Breeding, Volume I: Biotic Stress. Chichester: John Wiley & Sons; 2013. p. 151–182

19. Nabi A, Lateef I, Nisa Q, Banoo A, Rasool RS, Shah MD, et al. Phaseolus vulgaris−Colletotrichum lindemuthianum pathosystem in the post−genomic era: an update. Curr. Microbiol. 2022; 79: 36.

~~18.~~20. Banoo A, Nabi A, Rasool RS, Shah MD, Ahmad M, Sofi PA, Itoo H, Sharma PN, Padder BA. North-western Himalayan common beans: population structure and mapping of quantitative anthracnose resistance through genome wide association study. Front Plant Sci. 2020; 11: 571618.

~~19.~~21. Geffroy V, Sicard D, de Oliveira JC, Sévignac M, Cohen S, Gepts P, et al. Identification of an ancestral resistance gene cluster involved in the coevolution process between *Phaseolus vulgaris* and its fungal pathogen *Colletotrichum lindemuthianum*. Mol Plant Microbe Interact. 1999; 12(9): 774-784.

~~20.~~22. Gonçalves-Vidigal MC, Lacanallo GF, Vidigal PS. A new Andean gene conferring resistance to anthracnose in common bean (*Phaseolus vulgaris* L.) cultivar Jalo Vermelho. Plant Breed. 2008; 127: 592–596.

~~21.~~23. Gonçalves-Vidigal MC, Vidigal Filho PS, Medeiros AF, Pastor-Corrales MA. Common bean landrace Jalo Listras Pretas is the source of a new Andean anthracnose resistance gene. Crop Sci. 2009; 49: 133–138.

~~22.~~24. Gonçalves-Vidigal MC, Meirelles AC, Poletine JP, Sousa LL, Cruz AS, Nunes MP, et al. Genetic analysis of anthracnose resistance in 'Pitanga' dry bean cultivar. Plant Breed. 2012; 131: 423–429.

~~23.~~25. Oblessuc PR, Francisco C, Melotto M. The Co-4 locus on chromosome Pv08 contains a unique cluster of 18 COK-4 genes and is regulated by immune response in common bean. Theor Appl Genet. 2015; 128(6): 1193-1208.

~~24.~~26. Campa A, Trabanco N, Ferreira JJ. Identification of clusters that condition resistance to anthracnose in the common bean differential cultivars AB136 and MDRK. Phytopathology. 2017; 107(12): 1515-1521.

Formatted: Font: (Default) Times New Roman

~~25.~~27. Zuiderveen GH, Padder BA, Kamfwa K, Song Q, Kelly JD. Genome-wide association study of anthracnose resistance in Andean beans. PLoS ONE. 2016; 11: e0156391.

~~26.~~28. Padder BA, Sharma PN, Awale HE, Kelly JD. *Colletotrichum lindemuthianum*, the causal agent of bean anthracnose. Plant Pathol. 2017; 317-330

~~27.~~29. Pastor-Corrales MA. Estandarización de variedades diferenciales y de designación de razas de *Colletotrichum lindemuthianum*. Phytopathology. 1991; 81(6): 694.

30. Mungalu H, Sansala M, Hamabwe S, Mukuma C, Gepts P, Kelly JD, et al. Identification of race-specific quantitative trait loci for resistance to *Colletotrichum lindemuthianum* in an Andean population of common bean. Crop Sci. 2020; 60(6): 2843-2856.

31. Campa A, Rodríguez-Suárez C, Giraldez R, Ferreira JJ. Genetic analysis of the response to eleven Colletotrichum lindemuthianum races in a RIL population of common bean (Phaseolus vulgaris L.). BMC Plant Biology. 2014; 14:115.

32. González AM, Yuste-Lisbona FJ, Rodiño AP, De Ron AM, Capel C, García-Alcázar M, Santalla M. Uncovering the genetic architecture of Colletotrichum lindemuthianum resistance through QTL mapping and epistatic interaction analysis in common bean. Front Plant Sci. 2015; 6: 141.

~~28.~~33. Murube E, Campa A, Ferreira JJ. Integrating genetic and physical positions of the anthracnose resistance genes described in bean chromosomes Pv01 and Pv04. PLoS ONE. 2019; 14: e0212298.

Formatted: List Paragraph, Indent: Left: -0.05", Line spacing: Double, Numbered + Level: 1 + Numbering Style: 1, 2, 3, … + Start at: 1 + Alignment: Left + Aligned at: -0.5" + Indent at: -0.25"

Formatted: Font: (Default) Times New Roman

~~29.~~34. Balardin RS, Jarosz AM, Kelly JD. Virulence and molecular diversity in *Colletotrichum lindemuthianum* from South, Central, and North America. Phytopathology. 1997; 87(12): 1184-1191

~~30.~~35. SAS Institute. SAS version 9.3. SAS Institute Inc., Cary, NC. 2011.

~~31.~~36. Bradbury PJ, Zhang Z, Kroon DE, Casstevens TM, Ramdoss Y, Buckler ES. TASSEL: software for association mapping of complex traits in diverse samples. Bioinformatics. 2007; 23(19): 2633-2635.

~~32.~~37. Zhang Z, Ersoz E, Lai CQ, Todhunter RJ, Tiwari HK, Gore MA, et al. Mixed linear model approach adapted for genome-wide association studies. Nat Genet. 2010; 42(4): 355-360.

~~33.~~38. Schmutz J, McClean PE, Mamidi S, Wu GA, Cannon SB, Grimwood J, et al. A reference genome for common bean and genome-wide analysis of dual domestications. Nat Genet. 2014; 46(7): 707-713

~~34.~~39. Kamfwa K, Cichy KA, Kelly JD. Genome-wide association analysis of symbiotic nitrogen fixation in common bean. Theor Appl Genet. 2015; 128(10): 1999-2017.

40. Mahiya-Farooq, Padder BA, Bhat NN, Shah MD, Shikari AB, Awale HE, et al. Temporal expression of candidate genes at the Co-1 locus and their interaction with other defense related genes in common bean. Physiol Mol Plant Pathol. 2019; 108: 101424

~~35.~~41. Lima LR, Gonçalves-Vidigal MC, Vaz Bisneta M, Valentini G, Vidigal Filho PS, Martins VD, de Souza TL. Genetic fine-mapping of anthracnose disease-resistance allele Co-14 present in the Andean common bean cultivar AND 277. Crop Science. 2023;63(2):750-63.

Formatted: List Paragraph, Indent: Left: -0.05", Line spacing: Double, Numbered + Level: 1 + Numbering Style: 1, 2, 3, … + Start at: 1 + Alignment: Left + Aligned at: -0.5" + Indent at: -0.25"

Formatted: Font: (Default) Times New Roman, Font color: Custom Color(RGB(34,34,34))

Formatted: Font: Font color: Custom Color(RGB(34,34,34))

Formatted: Font: (Default) Times New Roman, Font color: Custom Color(RGB(34,34,34))

Formatted: Font: Font color: Custom Color(RGB(34,34,34))

Formatted: Font: (Default) Times New Roman, Font color: Custom Color(RGB(34,34,34))

Formatted: Font: Font color: Custom Color(RGB(34,34,34))

Formatted: Font: (Default) Times New Roman, Font color: Custom Color(RGB(34,34,34))

Formatted: Font: Font color: Custom Color(RGB(34,34,34))

Formatted: Font: (Default) Times New Roman, Font color: Custom Color(RGB(34,34,34))

Formatted: Font: (Default) Times New Roman

~~36.~~42. Valentini G, Goncalves-Vidigal M, Cregan P, Song Q, Pastor-Corrales MA. High-resolution mapping reveals linkage between genes in common bean cultivar Ouro Negro conferring resistance to the rust, anthracnose, and angular leaf spot diseases. Theor Appl Genet. 2017; 130: 1705-1722.

~~37.~~43. Binagwa PH, Traore SM, Egnin M, Bernard GC, Ritte I, Mortley D, et al. Genome-Wide Identification of Powdery Mildew Resistance in Common Bean (*Phaseolus vulgaris* L.). Front Genet. 2021; 12: 673069.

~~38.~~44. Tock AJ, Fourie D, Walley PG, Holub EB, Soler A, Cichy KA, et al. Genome-wide linkage and association mapping of halo blight resistance in common bean to race 6 of the globally important bacterial pathogen. Front Plant Sci. 2017; 7(8): 1170.

Supporting information

S1 Table. Severity scores for races 5, 19, 39, 51, 81, 183, 1050 and 1105 of *C. lindemuthianum* for the 255 Yellow Bean Collection genotypes evaluated in the greenhouse at University of Zambia, Lusaka, Zambia

Responses to Reviewer Comments

Reviewer # 1

Comment: The work done in this study refers to a very important biotic constraint in common bean production and some useful information has been generated in this study but authors have failed to signify the importance and novelty of this work as the MS resistance has been written as routine presentation of the data generated in the study.
Response: This is the first Genome-wide Association Study (GWAS) on common bean anthracnose conducted using the yellow bean diversity panel. The study has provided important and novel information on the genetic architecture of anthracnose resistance in yellow beans, which is a major market class of common bean. In addition to confirming previously identified genomic regions for anthracnose (ANTH) resistance, the study has reported novel genomic regions for ANTH resistance. The genomic regions on chromosomes Pv05 and Pv07 that provided resistance to race 39 are novel and reported for the first time in this manuscript. The other novelty is in the races used in the current study. For the majority of these races, it is the first time the genomic regions providing resistance to them has been established, and clusters of resistance genes associated with these specific races identified. Furthermore, this study has identified a yellow bean genotype YBC278 that is highly resistant to ANTH. This is a key finding with potentially major impact on the bean breeding community working on developing yellow bean varieties resistant to ANTH. There is no previous report of a yellow bean genotype that has higher ANTH resistance than YBC278 identified in the current study. In addition to the "standard" presentation of GWAS results in the form of Manhattan plots and tables showing genomic regions associated with resistance used in the current, the manuscript has gone further to better understand the positional candidate genes underlying the significant genomic regions. The study has determined that some of the positional candidate genes underlying the identified resistance loci occur in clusters, which could possibly explain why some of these resistance loci provide resistance to multiple races of *C. lindemuthianum*.

Comment: The methodology details of this work are detailed in reference 4 (Genet Resour Crop Evol. 2022; 69(4): 1627-1648) cited in this study. Only citation of reference to qualify the methodology used is not sufficient to help the reader and signify the work done, hence a gist of the protocol or procedures used need mention in the material and method section.
Response: We have revised the sections, Plant materials, Genotypic data analyses and Population structure under Materials and Methods that hopefully addresses this comment.

Comment: The presentation of the data in figures is very complex and is not able to access the descriptions of the results, hence need proper attention to depict the results in figures.
Response: Results for this GWAS have been presented in a "standard" way, which involves use of histograms and tables to summarize the phenotypic data, and identify patterns in the data. Further, GWAS results have been presented using Manhattan plots and tables to show genomic regions associated with resistance to the eight races used in

the current study. These tables, histograms and Manhattan plots were used to ensure that presentations of results are not complicated, and followed a “standard” way of presenting GWAS results.

Comment: Almost more than 95% of the genotypes were susceptible to most of the races. it's not clear that what were the basis of selecting the panel of genotype in this study need clarity.
Response: A number of considerations went into assembling the panel and its choice for use in the current study. First, the panel was assembled to specifically focus on yellow beans. The second consideration was that the panel had to have rich diversity of yellow bean genotypes and this was actualized by ensuring that the panel was comprised of yellow bean genotypes from breeding programs from different parts of the world. Yes, 95% of materials might have been susceptible to most races, but genotypes (few) with superior resistance (Table 2) comparable to that of even the resistant check for some races were identified. This demonstrated the importance and novelty of the study, because the identified resistant genotypes, few as they may be, are crucially important as novel sources of resistance to breed for improved ANTH resistance in yellow bean.

Comment: There are few more observations in the reviewed copy of the MS, which can improve the quality of the work done for better resolution of the results and facilitate the reader to come to conclusion of the study.
Response: Thank you for the observations. The manuscript has been revised to include all the observations that were in the reviewed copy of the manuscript.

Comment: The results are not properly described. There is no mention of the SNPs that can help in future study and likewise other output issues.
Response: We have revised the Results section to include the name of the SNPs associated with resistance to races used in the current study. The SNPs are mentioned on pages 17, 18 and 19 under Results. Furthermore, the SNP names and positions have been provided in Table 3. The reason we did not provide the SNP names initially was because we thought presenting positions or genomic regions significantly associated with resistance was more useful and could facilitate future comparisons of genomic regions identified in the current study with future studies. Names of SNPs do change depending on the genotyping platform used, but the positions do not change or change very slightly when there is a new version of the genome.

Reviewer # 2
Comment: The manuscript is well written and finding a few novel anthracnose associated SNPs on PV 05 and Pv07 are of interest.
Response: Thank you.

Comment: There are a few typos that need due attention and are listed below
Response: The typo’s have been corrected.

Comment: A few important references are missing from the manuscript.

Response: Several missing key references have been added to the revised manuscript.

Comment: L68-69. There is typo. Delete of and insert, hey?
Response: It was a typo and it has been corrected.

Comment: L86. ANTH may be replaced with anthracnose
Response: Replaced.

Comment: L89-91. Recently Nabi et al., 2022 have compiled this information. Not few QRLs now many have been identified do check (Simson et al., 2022; Banoo et al., 2020).
Response: Corrected and the three references have been added.

Comment: L111-112. Please do rewrite it.
Response: The sentence has been rewritten.

Comment: L117. Yellow bean Collection (YBC). Do write this acronym before also and define it in the first paragraph.
Response: Corrected

Comment: L132-134. Do rewrite it
Response: The sentence has been rewritten.

Comment: L135. Delete effect.
Response: Corrected.

Comment: L155 and L166 [28] may be replaced with Mungalu et al., (2020) as author year style.
Response: Corrected.

Comment: L168-169. Sub-script the magnesium sulphate and potassium dihydrogen othrophosphate
Response: Corrected.

Comment: L176 do provide reference at the end
Response: The reference has been added.

Comment: L206 and 212 [4] write the reference in author (year) style
Response: The author style has been changed.

Comment: L435. Do add a recent reference (Lima et al., 2023) that validated this gene model for anthracnose resistance. There are numerous GWAS studies that indicate association of this region with multiple anthracnose resistance. Do add write of such reference to support your conclusion
Response: The suggested reference has been added.

Comment: L467. Add a few more references

Response: References have been added.

Comment: L492. Banoo et al also found SNP on PV05 and 07 against race 87 and 73. Do add that also.
Response: The suggested reference has been added.
