# Supplementary material for: Genome-wide association analysis of anthracnose resistance in the Yellow Bean Collection of Common Bean
Source: PLoS One. 2023 Nov 10;18(11):e0293291. doi: 10.1371/journal.pone.0293291 (PMC10637669; doi:10.1371/journal.pone.0293291)
Supplement: Attachment — Submitted filename: Response to Reviewers.docx [file pone.0293291.s003.docx]

**Responses to Reviewer Comments**

**Reviewer # 1**

**Comment:** The work done in this study refers to a very important biotic constraint in common bean production and some useful information has been generated in this study but authors have failed to signify the importance and novelty of this work as the MS resistance has been written as routine presentation of the data generated in the study.

**Response:** This is the first Genome-wide Association Study (GWAS) on common bean anthracnose conducted using the yellow bean diversity panel. The study has provided important and novel information on the genetic architecture of anthracnose resistance in yellow beans, which is a major market class of common bean. In addition to confirming previously identified genomic regions for anthracnose (ANTH) resistance, the study has reported novel genomic regions for ANTH resistance. The genomic regions on chromosomes Pv05 and Pv07 that provided resistance to race 39 are novel and reported for the first time in this manuscript. The other novelty is in the races used in the current study. For the majority of these races, it is the first time the genomic regions providing resistance to them has been established, and clusters of resistance genes associated with these specific races identified. Furthermore, this study has identified a yellow bean genotype YBC278 that is highly resistant to ANTH. This is a key finding with potentially major impact on the bean breeding community working on developing yellow bean varieties resistant to ANTH. There is no previous report of a yellow bean genotype that has higher ANTH resistance than YBC278 identified in the current study. In addition to the “standard” presentation of GWAS results in the form of Manhattan plots and tables showing genomic regions associated with resistance used in the current, the manuscript has gone further to better understand the positional candidate genes underlying the significant genomic regions. The study has determined that some of the positional candidate genes underlying the identified resistance loci occur in clusters, which could possibly explain why some of these resistance loci provide resistance to multiple races of *C. lindemuthianum*.

**Comment:** The methodology details of this work are detailed in reference 4 (Genet Resour Crop Evol. 2022; 69(4): 1627-1648) cited in this study. Only citation of reference to qualify the methodology used is not sufficient to help the reader and signify the work done, hence a gist of the protocol or procedures used need mention in the material and method section.

**Response:** We have revised the sections, Plant materials, Genotypic data analyses and Population structure under Materials and Methods that hopefully addresses this comment.

**Comment:** The presentation of the data in figures is very complex and is not able to access the descriptions of the results, hence need proper attention to depict the results in figures.

**Response:** Results for this GWAS have been presented in a “standard” way, which involves use of histograms and tables to summarize the phenotypic data, and identify patterns in the data. Further, GWAS results have been presented using Manhattan plots and tables to show genomic regions associated with resistance to the eight races used in the current study. These tables, histograms and Manhattan plots were used to ensure that presentations of results are not complicated, and followed a “standard” way of presenting GWAS results.

**Comment:** Almost more than 95% of the genotypes were susceptible to most of the races. it's not clear that what were the basis of selecting the panel of genotype in this study need clarity.

**Response**: A number of considerations went into assembling the panel and its choice for use in the current study. First, the panel was assembled to specifically focus on yellow beans. The second consideration was that the panel had to have rich diversity of yellow bean genotypes and this was actualized by ensuring that the panel was comprised of yellow bean genotypes from breeding programs from different parts of the world. Yes, 95% of materials might have been susceptible to most races, but genotypes (few) with superior resistance (Table 2) comparable to that of even the resistant check for some races were identified. This demonstrated the importance and novelty of the study, because the identified resistant genotypes, few as they may be, are crucially important as novel sources of resistance to breed for improved ANTH resistance in yellow bean.

**Comment:** There are few more observations in the reviewed copy of the MS, which can improve the quality of the work done for better resolution of the results and facilitate the reader to come to conclusion of the study.

**Response:** Thank you for the observations. The manuscript has been revised to include all the observations that were in the reviewed copy of the manuscript.

**Comment:** The results are not properly described. There is no mention of the SNPs that can help in future study and likewise other output issues.

**Response:** We have revised the Results section to include the name of the SNPs associated with resistance to races used in the current study. The SNPs are mentioned on pages 17, 18 and 19 under Results. Furthermore, the SNP names and positions have been provided in Table 3. The reason we did not provide the SNP names initially was because we thought presenting positions or genomic regions significantly associated with resistance was more useful and could facilitate future comparisons of genomic regions identified in the current study with future studies. Names of SNPs do change depending on the genotyping platform used, but the positions do not change or change very slightly when there is a new version of the genome.

**Reviewer # 2**

**Comment:** The manuscript is well written and finding a few novel anthracnose associated SNPs on PV 05 and Pv07 are of interest.

**Response:** Thank you.

**Comment:** There are a few typos that need due attention and are listed below

**Response:** The typo’s have been corrected.

**Comment:** A few important references are missing from the manuscript.

**Response:** Several missing key references have been added to the revised manuscript.

**Comment:** L68-69. There is typo. Delete of and insert, hey?

**Response:** It was a typo and it has been corrected.

**Comment:** L86. ANTH may be replaced with anthracnose

**Response:** Replaced.

**Comment:** L89-91. Recently Nabi et al., 2022 have compiled this information. Not few QRLs now many have been identified do check (Simson et al., 2022; Banoo et al., 2020).

**Response:** Corrected and the three references have been added.

**Comment:** L111-112. Please do rewrite it.

**Response:** The sentence has been rewritten.

**Comment:** L117. Yellow bean Collection (YBC). Do write this acronym before also and define it in the first paragraph.

**Response:** Corrected

**Comment:** L132-134. Do rewrite it

**Response:** The sentence has been rewritten.

**Comment:** L135. Delete effect.

**Response:** Corrected.

**Comment:** L155 and L166 [28] may be replaced with Mungalu et al., (2020) as author year style.

**Response:** Corrected.

**Comment:** L168-169. Sub-script the magnesium sulphate and potassium dihydrogen othrophosphate

**Response:** Corrected.

**Comment:** L176 do provide reference at the end

**Response:** The reference has been added.

**Comment:** L206 and 212 [4] write the reference in author (year) style

**Response:** The author style has been changed.

**Comment:** L435. Do add a recent reference (Lima et al., 2023) that validated this gene model for anthracnose resistance. There are numerous GWAS studies that indicate association of this region with multiple anthracnose resistance. Do add write of such reference to support your conclusion

**Response:** The suggested reference has been added.

**Comment:** L467. Add a few more references

**Response:** References have been added.

**Comment:** L492. Banoo et al also found SNP on PV05 and 07 against race 87 and 73. Do add that also.

**Response:** The suggested reference has been added.
