# Supplementary material for: Genome-wide association analysis of anthracnose resistance in the Yellow Bean Collection of Common Bean
Source: PLoS One. 2023 Nov 10;18(11):e0293291. doi: 10.1371/journal.pone.0293291 (PMC10637669; doi:10.1371/journal.pone.0293291)
Supplement: Attachment — Submitted filename: Response to Reviewers.docx [file pone.0293291.s005.docx]

**Responses to Reviewer Comments**

**Reviewer # 1**

**Comments:** The authors have made very good attempt to improve the MS and now seems to be in order, however, still there are some queries and observations that need quick attention so that the MS could be processed further.

**Response:** The queries and observations have been worked on.

**Comment:** The author must clearly state that this is a unique panel not used earlier, need proper mention.

**Response:** A sentence has been included in both the abstract and introduction that the Yellow Bean Collection has not been previously used in genetic studies.

**Comment:** Page 8, line182-186, Dear Author, see the ref 34, as per this plant showing reaction 1-3 are considered resistant, whereas plant showing reaction 4-9 are considered as susceptible, so please clarify how you have rated score 4-6 as MR, need proper explanation.

**Response:** The severity scores in ref 34 (Balardin et al.) were on a scale 1-9. The reason they classified 4-9 as susceptible was because that paper was on characterization of C. lindemuthianum, and a final binary score of either resistant or susceptible is required. However, in breeding we have three classifications (resistant, moderately resistant and susceptible). Scores of 4-6 are classified as moderately resistant. We have replaced Balardin et al. with a more appropriate citation (van Schoonhoven and Pastor-Corrales) to avoid ambiguity.

**Comment:** Page 28, line no 414-415, check the statement and correct

**Response:** Statement corrected

**Comments:** There are some typographical and grammatical errors marked in the reviewed copy, be taken care of to improve the MS quality

**Response:** The typographical and grammatical errors that were highlighted in the reviewed copy have all been corrected.

**Reviewer #2**

**Comment:** There are a few typos that need due attention

**Response:** We have corrected all the typos that were noticed.

**Comment:** L98-99. Please check the sentencse -----genomic?. Position or region must be included

**Response:** The sentence has been corrected and physical position has also been provided.

**Comment:** L 104. There are about 298 races now reported in C. lindemuthianum (Nunes et al., 2021), 182 races were previously identified (Padder et al., 2017).

**Response:** The correction has been made and the suggested up-to-date reference (Nines et al., 2021) has been included.

**Comment:** L132. Delete “GWAS using”

**Response:** Corrected

**Comment:** L175. The author (year) is included but do check Plosone reference style in such a case.

**Response:** Corrected to be compliant with PLoS ONE reference style.

**Comment:** L301. Delete one that. That is written twice

**Response:** Corrected

**Comment:** L385. (Figu) or (Fig.)

**Response:** Corrected

**Comment:** L494. Banoo et al., 2020. Please see reference author (year) style of Plosone.

**Response:** Corrected to be compliant with PLoS ONE reference style.
